# Complete Chloroplast Genome of *Cercis chuniana* (Fabaceae) with Structural and Genetic Comparison to Six Species in Caesalpinioideae

**DOI:** 10.3390/ijms19051286

**Published:** 2018-04-25

**Authors:** Wanzhen Liu, Hanghui Kong, Juan Zhou, Peter W. Fritsch, Gang Hao, Wei Gong

**Affiliations:** 1 College of Life Sciences, South China Agricultural University, Guangzhou 510614, China; xixuegui17@163.com (W.L.); zj13808847221@126.com (J.Z.); 2 Key Laboratory of Plant Resources Conservation and Sustainable Utilization, South China Botanical Garden, Chinese Academy of Sciences, Guangzhou 510650, China; konghh@scbg.ac.cn; 3 Guangdong Provincial Key Laboratory of Applied Botany, South China Botanical Garden, Chinese Academy of Sciences, Guangzhou 510650, China; 4 Botanical Research Institute of Texas, 1700 University Drive, Fort Worth, TX 76107, USA; pfritsch@brit.org

**Keywords:** *Cercis chuniana*, Cercidoideae, Caesalpinioideae, chloroplast genome, legume, next-generation sequencing

## Abstract

The subfamily Caesalpinioideae of the Fabaceae has long been recognized as non-monophyletic due to its controversial phylogenetic relationships. *Cercis chuniana*, endemic to China, is a representative species of *Cercis* L. placed within Caesalpinioideae in the older sense. Here, we report the whole chloroplast (cp) genome of *C. chuniana* and compare it to six other species from the Caesalpinioideae. Comparative analyses of gene synteny and simple sequence repeats (SSRs), as well as estimation of nucleotide diversity, the relative ratios of synonymous and nonsynonymous substitutions (dn/ds), and Kimura 2-parameter (K2P) interspecific genetic distances, were all conducted. The whole cp genome of *C. chuniana* was found to be 158,433 bp long with a total of 114 genes, 81 of which code for proteins. Nucleotide substitutions and length variation are present, particularly at the boundaries among large single copy (LSC), inverted repeat (IR) and small single copy (SSC) regions. Nucleotide diversity among all species was estimated to be 0.03, the average dn/ds ratio 0.3177, and the average K2P value 0.0372. Ninety-one SSRs were identified in *C. chuniana*, with the highest proportion in the LSC region. Ninety-seven species from the old Caesalpinioideae were selected for phylogenetic reconstruction, the analysis of which strongly supports the monophyly of Cercidoideae based on the new classification of the Fabaceae. Our study provides genomic information for further phylogenetic reconstruction and biogeographic inference of *Cercis* and other legume species.

## 1. Introduction

The chloroplast (cp) is widely present in algae and plants with important functions in photosynthesis, carbon fixation, and stress response [1,2]. The cp genome in most angiosperms is a circular molecule with a typically quadripartite structure, comprising a large single copy (LSC) region and a small single copy (SSC) region separated by two copies of a large inverted repeat (IR) region [3,4,5,6]. Although the cp genome is highly conserved, some differences in gene synteny, simple sequence repeats (SSRs) and pseudogenes have been observed [7,8,9] and an accelerated rate of evolution has been observed in some cp regions at different taxonomic levels [10,11]. A complete cp genome is a valuable resource of information for studying plant taxonomy, phylogenetic reconstruction, and historical biogeographic inference. Next-generation sequencing (NGS) technologies have enabled a rapid expansion in the database of whole cp genomes [12,13].

Fabaceae (legumes) are the third largest angiosperm family, with an estimated 727 genera and 20,000 species [14]. The family has been traditionally classified into three well-known and widely accepted subfamilies, i.e., Caesalpinioideae DC., Mimosoideae DC. and Papilionoideae DC. However, the subfamily Caesalpinioideae has been long considered to be non-monophyletic and not reflective of accurate phylogenetic relationships among the species [15,16,17,18,19,20]. As based on recent phylogenetic analyses, a new classification of six subfamilies has been recognized in Leguminosae: Cercidoideae, Detarioideae, Dialioideae, Duparquetioideae, Papilionoideae and a recircumscribed Caesalpinioideae [21].

The genus *Cercis* L. is removed from Caesalpinioideae and currently placed within Cercidoideae [21]. This genus comprises a clade of about nine species, with a disjunct distribution across the warm temperate zones of Eastern Asia, Europe and North America [22,23,24,25,26]. In China, five species are recognized, i.e., *C. chinensis*, *C. chingii*, *C. chuniana*, *C. glabra* and *C. racemosa* [26,27]. *Cercis chuniana*, a small tree or shrub, occurs mainly in subtropical evergreen broadleaf forest with a relatively narrow geographic distribution in southern China. Unique among *Cercis* species, it has an asymmetrical leaf blade [27,28].

Previous research has been focused on plant anatomy, phylogenetic reconstruction, and historical biogeography of *Cercis* [24,25,26,29,30]. However, *C. chuniana* has frequently failed to be analyzed in most phylogenetic research, resulting in an unclear phylogenetic position within the genus. Because *Cercis* has been removed to Cercidoideae, it would also be useful to detect additional genomic evidence that might support the new classification system of Fabaceae. Moreover, Sanger-based and whole-cp genome DNA barcoding can been used for phylogenetic reconstruction. Here we present and characterize the complete cp genome of *C. chuniana*. The structural variation, gene arrangement, and distribution of SSRs are compared with previously published cp genome of *C. canadensis* and five species from various genera in Caesalpinioideae. Our results provide cp information for *Cercis* and other legumes for use in comparative genomics, phylogenetic reconstruction, and biogeographic inference.

## 2. Results and Discussion

### 2.1. Genome Organization and Features of C. chuniana

**Figure 1:**
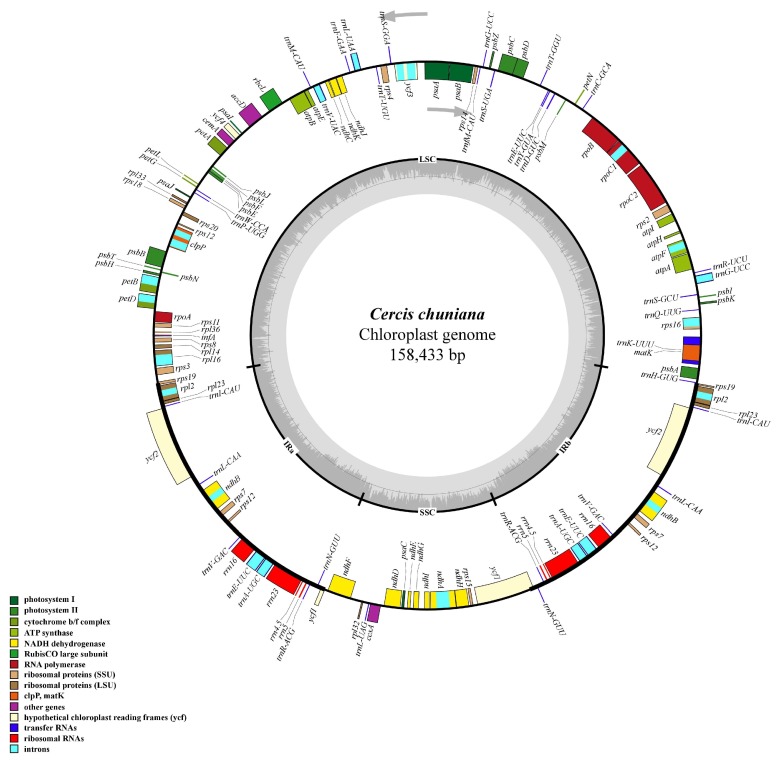
Gene map of the *Cercis chuniana* cp genome. The genes lying inside and outside the outer circle are transcribed in clockwise and counterclockwise direction, respectively (as indicated by arrows). Colors denote the genes belonging to different functional groups. The hatch marks on the inner circle indicate the extent of the inverted repeats (IRa and IRb) that separate the small single copy (SSC) region from the large single copy (LSC) region. The dark gray and light gray shading within the inner circle correspond to percentage G + C and A + T content, respectively.

A total number of 2 × 250 bp pair-end reads of 1,917,920 were produced with 1.17 Gb of clean data. All reads data were deposited in the NCBI Sequence Read Archive (SRA) under accession number SRP118607. In total, 102 contigs (N50 = 8438 bp) were generated for *C. chuniana*. The size of the complete cp genome is 158,433 bp (Figure 1; Table 1). The cp genome displays a typical quadripartite structure, including a pair of IR regions (25,505 bp) separated by the LSC (88,063 bp) and SSC (19,360 bp) regions (Figure 1 and Table 1). The G + C content of the cp genome is 36.10% for *C. chuniana*, demonstrating congruence with that of *C. canadensis* (36.20%) (Table 1). When duplicated genes in the IR regions were counted only once, the cp genome of *C. chuniana* were found to encode 114 predicted functional genes, including 81 protein-coding genes (PCGs), 29 tRNA genes, and four rRNA genes, all of which are comparable to the numbers in *C. canadensis* and other related species (Table 1). The remaining non-coding regions include introns, intergenic spacers, and pseudogenes. Nineteen genes are duplicated in the IR regions, including eight PCGs, seven tRNA genes, and four rRNA genes (Figure 1 and Table S1). Fifteen genes (nine PCGs and six tRNA genes) contain one intron, and two PCGs (*clpP* and *ycf3*) have two introns each (Table S1). The maturase K (*matK*) gene in the cp genome is located within the *trnK* intron, consistent with the location in *C. canadensis* and similar to most other plant species [31]. In the IR regions of *C. chuniana*, the four rRNA genes and two tRNA genes (*trnE* and *trnA*) are clustered as 16S-*trnE*-*trnA*-23S-4.5S-5S. This differs from the cp genomes of *C. canadensis* and most legumes, which show a cluster of 16S-*trnI*-*trnA*-23S-4.5S-5S [32,33,34,35,36,37].

### 2.2. Comparative Analysis of Genomic Structure

**Figure 2:**
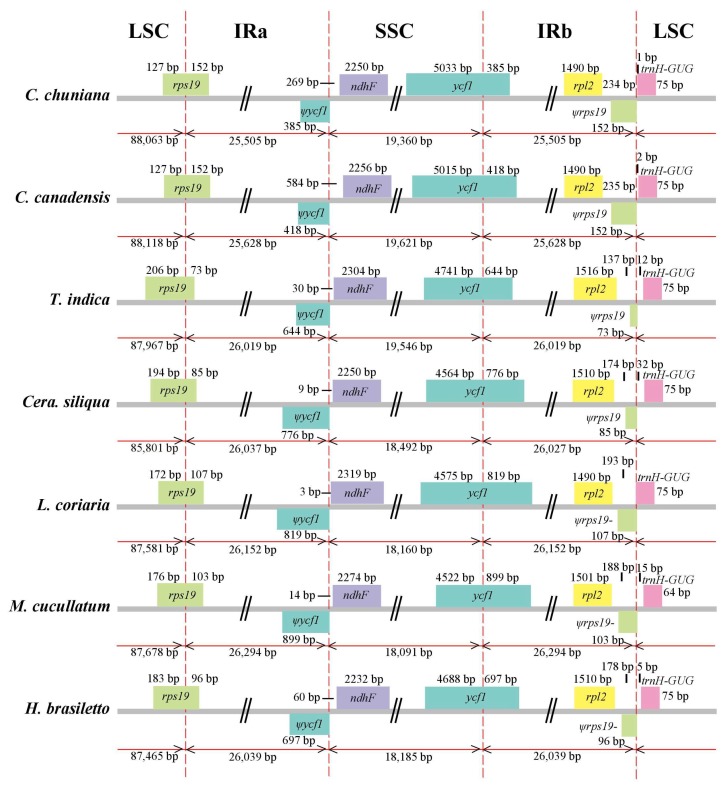
Comparison of the border positions of LSC, SSC and IR regions among the seven species of caesalpinioid legumes compared in this study. Genes are denoted by colored boxes. The gaps between the genes and the boundaries are indicated by the base lengths (bp). Extensions of the genes are indicated above the boxes.

Synteny analysis identified a lack of genome rearrangement and inversions in the cp genome sequences among the seven species (Figure S1). Therefore, genomic structure, including gene number and gene order, is highly conserved among the seven species. However, some nucleotide substitutions and indels as well as length variation are still present, particularly in the LSC/IR/SSC boundaries (Figure 2 and Figure S2).

Pseudogenes are frequently identified in cp genomes [38,39]. Four pseudogenes were identified in the current study, i.e., *Ψrps19*, *Ψycf1*, *ΨinfA* and *ΨaccD* (Table 2). *Ψrps19* and *ycf1* are partially repeated in the IR regions and were generally found to be pseudogenized. The *rps19* gene is 279 bp in all species (Figure 2) with length variation in the IR regions, from 73 bp in *Tamarindus indica* to 107 bp in *Libidibia coriaria*. It has the same length (152 bp) in both *C. chuniana* and *C. canadensis* in the IR regions (Figure 2). Because it is partially duplicated in the IR regions, the *Ψrps19* gene has lost its protein-coding ability, thus producing the pseudogenized *Ψrps*19 gene. Two nonsynonymous substitutions were detected in the *Ψrps19* gene between *C. chuniana* and *C. canadensis*. Among the seven species, 28 substitutions (seven in the IRb region and 21 in the LSC region, respectively) and 4 indels with length variation from 4 to 47 bp were identified (Figure 2; Table 2). The same was found with the *Ψycf1* gene, as the IRb/SSC junction region is located within the *Ψycf1* CDS region and only a partial gene is duplicated in the IRa region, thus producing the pseudogene *Ψycf1*. This is generally the case in the dicots. The length of the *Ψycf1* pseudogene in the IR regions ranges from 385 bp in *C. chuniana* to 899 bp in *Mezoneruon cucullatum*. Four nonsynonymous substitutions were detected between *C. chuniana* and *C. canadensis*. Altogether 20 substitutions (19 in the IRa region and one in the SSC region) and 7 indels with length variation ranging from 1 to 33 bp are present among the seven species (Figure 2; Table 2). The *ΨinfA* gene is pseudogenized in all species except *Ceratonia siliqua*, with a length of 135 bp in both *C. chuniana* and *C. canadensis* and with length ranging from 192 to 252 bp among the other four species. A total of 23 substitutions and 6 indels ranging from 1 to 13 bp in length occurs in *ΨinfA* (Figure 2; Table 2). The pseudogenized *ΨinfA* gene has also been frequently found in other angiosperm chloroplast genomes as well [40,41,42]. The pseudogenized *ΨaccD* gene is present in all species except *T. indica* and *M. cucullatum*, with a length of 1473 bp in both *C. chuniana* and *C. canadensis* and with length ranging from 1395 to 1500 bp in the other three species. Six indels ranging from 3 to 36 bp in length, and 101 substitutions were detected in *ΨaccD* (Table 2).

### 2.3. Characterization of Simple Sequence Repeats

**Figure 3:**
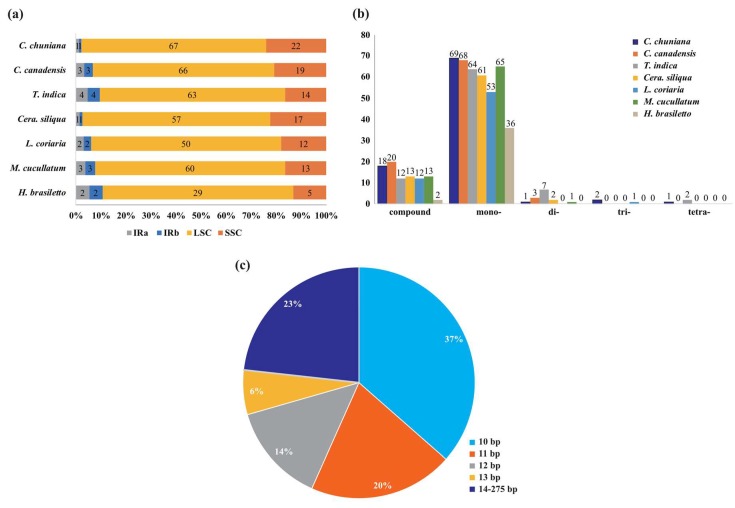
Analysis of repeated sequences of the seven species compared in this study. (**a**) The number of SSRs distributed in different regions; (**b**) The number of SSRs with different types, including compound, mono-, di-, tri-, and tetranucleotides; (**c**) The proportion of SSRs with different lengths.

Variable copy numbers and resulting length variation have impelled the wide use of cp SSRs in plant population genetics and biogeographic studies, especially at lower taxonomic levels [43,44]. A total of 91 SSRs of ≥10 bp in length were found in both *C. chuniana* and *C. canadensis*. These two species exhibit the highest number of SSRs among the seven species (Table 3). The lowest number of SSRs was detected in *Haematoxylum brasiletto*, with only 38 SSRs in total (Table 3). Most SSRs are present in the LSC regions, accounting for an average of 75.00% of the total SSRs in each species. Among all of the SSRs, the mononucleotide A + T repeat units were found in highest proportion, with an average of 78.10% of the total SSRs in each species. The SSRs have a remarkably high A or T content, with only 15 compound SSRs containing the nucleotides C or G in *C. chuniana* (Table S2). The lengths of SSRs in the seven species range from 10 to 20 bp, whereas the compound SSRs range from 21 to 275 bp. The copy lengths of 10 to 13 bp are most common, with an average of 77.00% among all species (Figure 3). No pentanucleotide or hexanucleotide SSRs were detected among the seven species.

The shared interspecific SSRs were identified among species, with identical repeats and locations in homologous regions (Table 4). *Cercis chuniana* and *C. canadensis* demonstrated the highest number of 19 common SSRs. Conversely, *Tamarindus indica* has the lowest number of shared SSRs (≤3). Altogether 13 SSRs were isolated and corresponding primer pairs were designed for each di-, tri- and tetranucleotide SSRs of *C. chuniana* (Table S3). These SSRs are expected to be useful in the assessment of genetic diversity and population structure as well as the investigations of biogeographic patterns among the species of *Cercis*.

### 2.4. Sequence Divergence and Nucleotide Diversity

**Figure 4:**
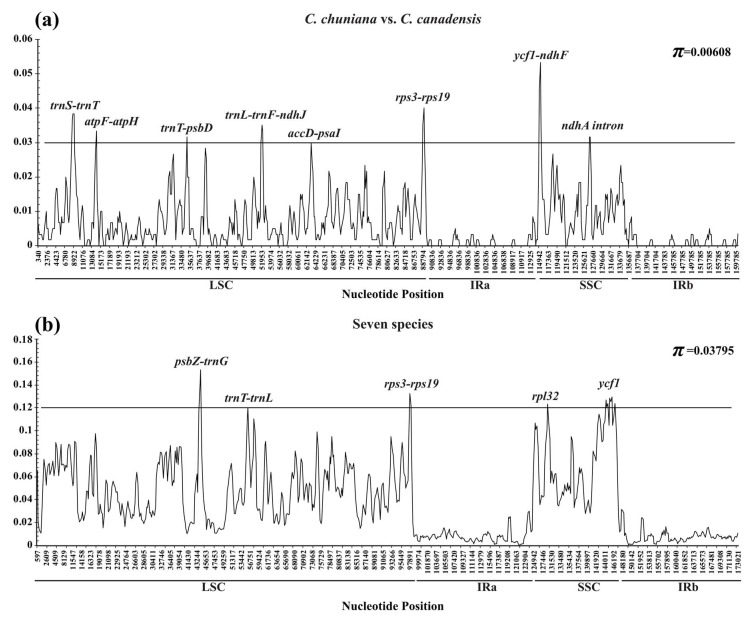
Sliding window analysis of the whole cp genome. (**a**) *C. chuniana* and *C. canadensis*; (**b**) All seven species. *X*-axis: position of the midpoint of a window; *Y*-axis: nucleotide diversity (π) of each window.

A complete cp genome is valuable for plant taxonomic analyses, phylogenetic reconstruction, speciation processes, and biogeographical inferences at different taxonomic levels [45,46,47,48,49]. Highly variable regions among cp genomes can provide useful data for phylogenetic reconstruction. In the current study, the average nucleotide variability (*Pi*) was estimated to be 0.006 between *C. chuniana* and *C. canadensis* as based on the comparative analysis with DnaSP (Figure 4a). The highest variation was found in the LSC and SSC regions. The IR regions had a much lower nucleotide diversity with *Pi* < 0.006. Eight regions (*trnS-trnT*, *atpF-atpH*, *trnT-psbD*, *trnL-trnF-ndhJ*, *accD-psaI*, *rps3-rps19*, *ycf1-ndhF* and the *ndhA* intron) were highly variable, with *Pi* values >0.030. The first five loci are present in the LSC, whereas the remaining two are present in the SSC region. In contrast, much higher nucleotide diversity with *Pi* = 0.038 was detected among the seven species (Figure 4b). Five regions (*psbZ-trnG*, *trnT-trnL*, *rps3-rps19*, *rpl32*, and *ycf1*) exhibit the highest nucleotide diversity, all with *Pi* >0.12. These loci are thus suggested as useful regions for phylogenetic analysis at higher taxonomic levels in the Fabaceae.

### 2.5. dn/ds Ratio and Kimura 2-Parameter (K2P) Genetic Distance

**Figure 5:**
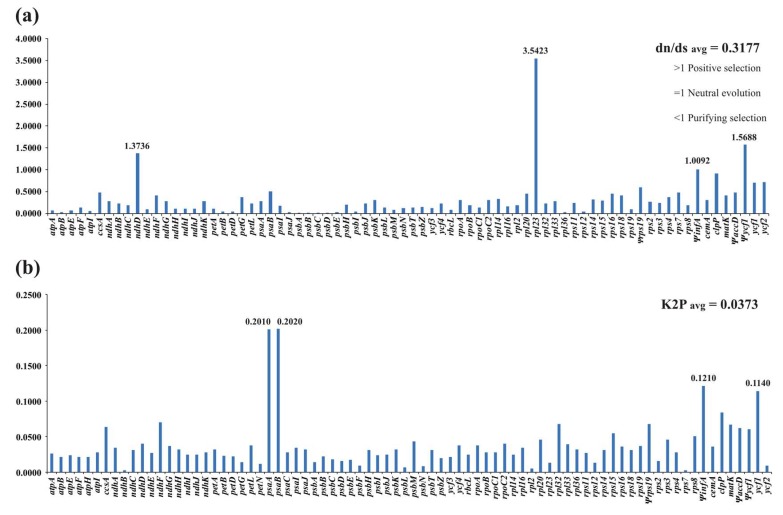
Evolutionary dynamics of genes in the cp genomes. (**a**) The dn/ds ratios for individual genes; (**b**) The K2P values for individual genes.

A total of 76 PCGs in all seven species was used to estimate dn/ds ratios. The dn and ds values range from 0 to 0.1713 and 0.0046 to 0.5330, respectively. If dn or ds is 0, the dn/ds ratio cannot be calculated. Among all genes, 67 proteins possess dn/ds ratios <0.5, indicating purifying selection (Figure 5a). In *ndhD*, *Ψycf1*, *ΨinfA* and *rpl23* the dn/ds ratios were >1, indicating positive selection (Figure 5a). Among the different regions, the dn/ds ratio was the highest in the IR regions (0.9022) and the lowest in the LSC region (0.2205). Based on the K2P model, we calculated the interspecific genetic distance among the seven species using 80 PCGs. The average K2P interspecific genetic distance was found to be 0.0373 (Figure 5b). The minimum K2P values were identified in *ndhB* and *rps7* (0.0030) and the maximum in *psaB* (0.2020).

### 2.6. Phylogenetic Analyses

**Figure 6:**
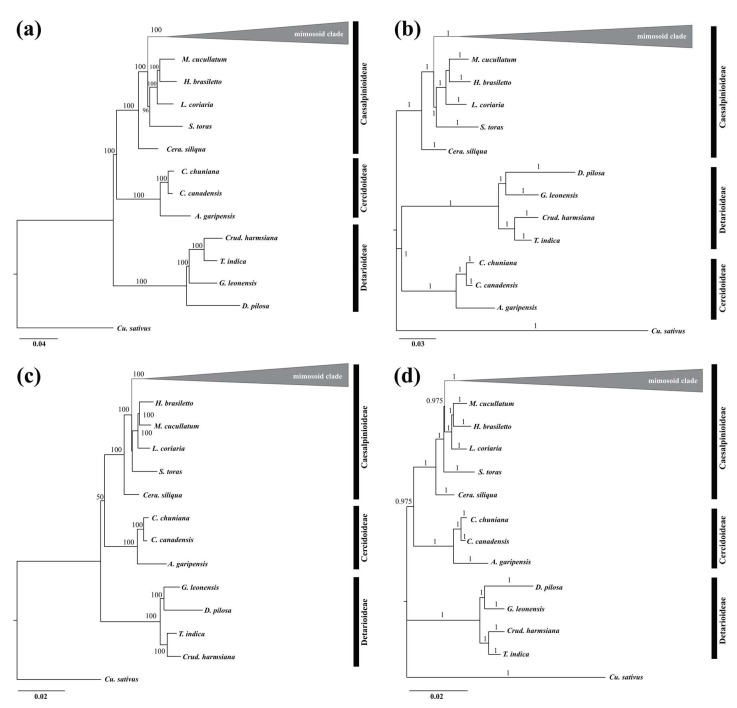
Phylogenetic trees of sampled species inferred from the concatenated whole cp genome sequences and 61 protein-coding genes (PCGs) in the cp genome based on maximum likelihood (ML) and Bayesian inference (BI). (**a**) ML analysis based on whole cp genome sequences; (**b**) BI analysis based on whole cp genome sequences; (**c**) ML analysis based on PCGs; (**d**) BI analysis based on PCGs. Numbers in bold above branches are bootstrap values ≥50% and Bayesian posterior probability values ≥90%.

A total of 97 representative species from the old Caesalpinioideae and Mimosoideae were selected to reconstruct phylogenetic relationships (Table S4). *Cucumis sativus* (DQ119058) was used as the outgroup. Two phylogenetic methods of Bayesian inference (BI) and maximum likelihood (ML) resulted in highly similar phylogenetic trees based on the complete cp genome sequences and 61 protein-coding genes (PCGs) (Figure 6). The total aligned length was 302,882 bp for the complete cp genome sequences and 69,253 bp for the PCGs, and the number of parsimony-informative sites was 163,470 bp and 25,698 bp, respectively. The trees based on ML exhibit completely congruent topologies with higher bootstrap support values in the tree based on complete cp genome sequences than those based on the PCGs (Figure 6a). The relationship between subfamilies Cercidoideae and Detarioideae was not stable in the BI analysis, but otherwise high posterior probability values were detected in both the ML and BI analyses based on the two data sets (Figure 6b). All analyses recover the monophyly of both the Cercidoideae and Detarioideae with strong support. Our results are consistent with [50] and strongly support the new classification system of the Fabaceae [21].

## 3. Materials and Methods

### 3.1. Ethics Statement

Sample collection and transplanting were carried out for scientific purposes. *Cercis chuniana* was collected from the field in Dadongshan Natural Reserve in Guangdong Province, China. One individual seedling was permitted by the management of the reserve to be transplanted and grown in the greenhouse at the College of Life Sciences, South China Agricultural University (SCAU, Guangzhou, China).

### 3.2. Plant Samples

Fresh leaves were collected from *C. chuniana* growing at SCAU. The voucher (LWZ109) is deposited in the herbarium of SCAU (CANT). The cp genome of *C. canadensis* (KF856619) was downloaded from NCBI and used as the reference sequence in the assembly of *C. chuniana*. Five additional species from the old Caesalpinioideae were used for comparison, i.e., *Tamarindus indica* (KJ468103), *Ceratonia siliqua* (KJ468096), *Libidibia coriaria* (KJ468095), *Mezoneuron cucullatum* (KU569489) and *Haematoxylum brasiletto* (KJ468097).

### 3.3. DNA Extraction and PCR Amplification

Total genomic DNA was extracted with the modified Cetyl Trimethyl Ammonium Bromide (CTAB) method [51]. The DNA concentration was quantified with a Nanodrop spectrophotometer (Thermo Scientific, Carlsbad, CA, USA), and a final DNA concentration of >30 ng/µL was used. Sequences of complete cp genome of *C. chuniana* were amplified with fifteen universal primer pairs developed by Zhang et al. [52]. The PCR amplification was performed in a total volume of 25 μL, containing 1 ng of template DNA, 0.12 U of Primerstar GXL DNA Polymerase, 0.2 μM of each primer, 200 μM of each dNTP, 5 μL of 5× PCR Buffer and 13.5 μL of sterilized double-distilled water. Thermocycling conditions were 95 °C (1 min), followed by 32 cycles of denaturation at 94 °C (15 s), annealing at 58 °C (30 s), and extension at 68 °C (10 min), and a final extension of 68 °C (10 min).

### 3.4. Chloroplast Genome Sequencing, Assembly and Annotation

A paired-end library was constructed with the Nextera XT DNA Library Prep Kit (Illumina Inc., San Diego, CA, USA). The genomic DNA mixture was fragmented into ~300 bp size by the Nextera XT transposome. Library Sequencing acquired 2 × 250 bp paired reads with Illumina MiSeq Desktop Sequencer at South China Botanical Garden, Chinese Academy of Sciences. Reads of the *C. chuniana* cp genome were initially filtered for quality, and then adapters were removed, errors were checked, and contigs and scaffolds generated, all with the A5-miseq pipeline [53]. Scaffolds from the assembly with *k*-mer values of 35 to 145 were matched to reference cp genome sequences, and were used to determine the relative position and direction respectively. We assembled the cp genome using Geneious 9.1.4 (Biomatters Ltd., Auckland, New Zealand) [54] with BLAST 2.0.3+ (National Institutes of Health, Bethesda, MD, USA) [55] and map reference tools. DOGMA (available online: http://dogma.ccbb.utexas.edu/) [56] and Geneious (Biomatters Ltd., Auckland, New Zealand) were used for annotating the cp genome in comparison with that of *C. canadensis* (KF856619) [57]. The annotation of tRNA genes were confirmed with the ARAGORN program (Lund University, Lund, Sweden) [58] and then manually adjusted with Geneious. Contigs with BLAST hits to the consensus sequence from the “map to reference function” were assembled manually to construct the complete cp genome. Finally, the circular genome map of *C. chuniana* was illustrated with the Organellar Genome DRAW tool (OGDRAW, available online: http://ogdraw.mpimp-golm.mpg.de/) [59]. To further refine the draft genome, the quality and coverage of was confirmed by remapping reads. The Sequence Read Archive (SRA) can be found in GenBank under an accession number of SRP118607. The annotated cp genomic sequence of *C. chuniana* was deposited in GenBank (Accession Number: MF741770).

### 3.5. Genome Comparison

The cp genome sequences from the finalized data set were aligned with MAFFT v7.0.0 (Osaka University, Suita, Japan) [60] and adjusted manually when necessary. The expansion/contraction of the IR regions can lead to changes in the structure of the cp genome, resulting in the length variation of angiosperm cp genomes and contributing to the formation of pseudogenes [9,61,62]. Therefore, we conducted a comparative analysis to detect the variation in the LSC/IR/SSC boundaries among the seven species included in comparisons. Gene synteny analysis was performed with MAUVE (University of Wisconsin, Madison, WI, USA) [63] as implemented in Geneious with default settings. To elucidate the level of sequence divergence, the complete cp genomes were compared and plotted with the mVISTA program in Shuffle-LAGAN mode [64,65,66].

### 3.6. Simple Sequence Repeats Analysis

MISA (available online: http://pgrc.ipk-gatersleben.de/misa/misa.html) [67] is a tool for the identification and location of perfect simple sequence repeat loci (SSRs) and compound SSRs (the latter being two individual SSRs that are disrupted by a certain number of bases). We used MISA to search for potential SSRs in the cp genomes of the seven species. The minimum number (thresholds) of SSRs was set as 10, 6, 5, 5, and 5 for mono-, di-, tri-, tetra-, and pentanucleotide SSRs, respectively. All SSRs, motif types and length variants were manually verified and the redundant ones removed. We investigated the shared repeats among the cp genomes of the seven species, based on the criterion that identical lengths located in homologous regions are considered to be shared repeats. Using the program Primer 3-1.1.1 (Premier Biosoft International, Palo Alto, CA, USA) [68], we developed SSR primers specific for *C. chuniana* for potential application in further analysis.

### 3.7. Sequence Divergence, dn/ds Ratio and K2P Genetic Distance

Comparative analyses of the nucleotide diversity (*Pi*) among the complete cp genomes of the seven species were performed with DnaSP 6 (Universitat de Barcelona, Barcelona, Spain) [69,70], as based on a sliding window analysis. The window length was 600 bp and step size was 200 bp. The 80 PCGs were extracted and aligned with MAFFT. We estimated the dn/ds ratio for each PCG as well as the interspecific genetic distance with DnaSP 6 and MEGA 6.0 (Tokyo Metropolitan University, Hachioji, Tokyo, Japan) [71], as based on the Kimura 2-parameter (K2P) model.

### 3.8. Phylogenetic Analysis

Altogether 97 representative species from the old Caesalpinioideae and Mimosoideae were selected for phylogenetic analyses (Table S4). *Cucumis sativus* (DQ119058) was used as the outgroup. Two data sets of the complete cp genome sequences and PCGs were used for phylogenetic reconstruction based on two methods of Bayesian inference (BI) and maximum likelihood (ML), respectively. All analyses were performed on the high-performance computer cluster available in the CIPRES Science Gateway 3.3 (available online: www.phylo.org) [72]. Gaps were treated as missing data. BI was performed by using MrBayes v. 3.2.6 (Swedish Museum of Natural History, Stockholm, Sweden) [73] with base frequencies estimated from the data. We ran four Markov Chains Monte Carlo (MCMC) for 50 million generations using default settings for priors and saved one tree every 1000 generations. The first 10% of the trees were discarded, as determined with the aid of the program Tracer version 1.6 (University of Auckland, Auckland, New Zealand) [74]. The posterior probability (PP) of each clade (i.e., the “clade credibility value”) was estimated with 50% majority-rule consensus trees. We conducted ML using RAxML 8.2.10 (Heidelberg Institute for Theoretical Studies, Heidelberg, Germany) [75] and the RAxML graphical interface (rxmlGUI v. 1.3) (Research Institute Senckenberg, Frankfurt, Germany) [76]. RaxML was conducted by using Python v.2.7.6 (available online: http://www.python.org/ftp/python/2.7.6/python-2.7.6.msi) with 1000 rapid bootstrap replicates. The general time-reversible (GTR) model was chosen with a gamma model for the rate of heterogeneity.

## 4. Conclusions

We report the complete cp genome of *C. chuniana* endemic to China, which belongs to *Cercis* L., an intercontinentally disjunct genus. Using a high-throughput sequencing method, we sequenced and annotated the whole genome, detected the arrangement of the genes, and identified SSRs in *C. chuniana*. We compared the cp genomic characteristics of *C. chuniana* to its congener *C. canadensis* and five other species from the old Caesalpinioideae. The current study is the first structural and gene comparison among the cp genomes of seven species from three subfamilies of legumes, including Cercidoideae, Detarioideae and Caesalpinioideae at the genomic level. Nearly 100 representative species from the old Caesalpinioideae and Mimosoideae were used for phylogenetic reconstruction, strongly corroborating the monophyly of Cercidoideae and Detarioideae in the sense of the new classification of Fabaceae. Our study contributes to the taxonomy, phylogenetic reconstruction and biogeographical research of *Cercis* and other legume species.

## Figures and Tables

**Table 1:** Summary of characteristics in cp genome sequences of *Cercis chuniana* and six other species of caesalpinioid legumes compared in this study.

Genome Features	*C. chuniana*	*C. canadensis*	*T. indica*	*Cera. siliqua*	*L. coriaria*	*M. cucullatum*	*H. brasiletto*
GenBank Accession No.	MF741770	KF856619	KJ468103	KJ468096	KJ468095	KU569489	KJ468097
Size (bp)	158,433	158,995	159,551	156,367	158,045	158,357	157,728
LSC length (bp)	88,063	88,118	87,967	85,801	87,581	87,663	87,465
SSC length (bp)	19,360	19,621	19,546	18,492	18,160	18,091	18,185
IR length (bp)	25,505	25,628	26,019	26,037	26,152	26,294	26,039
Number of genes	114	113	113	112	113	114	113
PCGs	81	79	79	78	80	80	79
tRNA genes	29	30	30	30	29	30	30
rRNA genes	4	4	4	4	4	4	4
G + C content (%)	36.10	36.20	36.20	36.70	36.50	36.40	36.70

**Table 2:** The location and characteristics of the four pseudogenes in the seven species of caesalpinioid legumes compared in this study.

Species	IRa	IRb	LSC	LSC
	*Ψycf1*	*Ψrps19*	*ΨinfA*	*ΨaccD*
*C. chuniana*	385 bp *	152 bp *	1 indel, 91-bp SV *	5 indels *
*C. canadensis*	418 bp *	152 bp *	1 indel, 91-bp SV *	5 indels *
*T. indica*	644 bp *	73 bp *	71-bp SV *	-
*Cera. siliqua*	776 bp *	85 bp *	-	4 indels, 63-bp SV *
*L. coriaria*	819 bp *	107 bp *	-	4 indels *
*M. cucullatum*	899 bp *	103 bp *	4 indels *	3 indels *
*H. brasiletto*	697 bp *	96 bp *	4 indels *	4 indels *

* Pseudogene present; SV: structural variation with indels ≥ 50 bp.

**Table 3:** Number of chloroplast SSRs in different regions or different types present in the seven species from caesalpinioid legumes.

Species	N	LSC	SSC	IRa	IRb	Compound	Mono- (≥10)	Di- (≥6)	Tri- (≥5)	Tetra- (≥5)
*C. chuniana*	91	67 (73.63)	22 (24.18)	1 (1.10)	1 (1.10)	18 (19.78)	69 (75.82)	1 (1.10)	2 (2.20)	1 (1.10)
*C. canadensis*	91	66 (72.53)	19 (20.88)	3 (3.30)	3 (3.30)	20 (21.98)	68 (74.73)	3 (3.30)	0 (0.00)	0 (0.00)
*T. indica*	85	63 (74.12)	14 (16.47)	4 (4.71)	4 (4.71)	12 (14.12)	64 (75.29)	7 (8.24)	0 (0.00)	2 (2.35)
*Cera. siliqua*	76	57 (75.00)	17 (22.37)	1 (1.32)	1 (1.32)	13 (17.11)	61 (80.26)	2 (2.63)	0 (0.00)	0 (0.00)
*L. coriaria*	66	50 (75.76)	12 (18.18)	2 (3.03)	2 (3.03)	12 (18.18)	53 (80.30)	0 (0.00)	1 (1.52)	0 (0.00)
*M. cucullatum*	79	60 (75.95)	13 (16.46)	3 (3.80)	3 (3.80)	13 (16.46)	65 (82.28)	1 (1.27)	0 (0.00)	0 (0.00)
*H. brasiletto*	38	29 (76.32)	5 (13.16)	2 (5.26)	2 (5.26)	2 (5.26)	36 (94.74)	0 (0.00)	0 (0.00)	0 (0.00)
Average	75	56 (74.76)	15 (18.81)	2 (3.22)	2 (3.22)	13 (17.14)	59 (79.24)	2 (2.67)	0.43 (0.57)	0.43 (0.57)

Note: The numbers in parentheses are the percentage of each region or SSR types.

**Table 4:** Shared SSRs among the seven species in caesalpinioid legumes.

Species	*C. chuniana*	*C. canadensis*	*T. indica*	*Cera. siliqua*	*L. coriaria*	*M. cucullatum*	*H. brasiletto*
*C. chuniana*	-						
*C. canadensis*	19	-					
*T. indica*	1	0	-				
*Cera. siliqua*	6	4	1	-			
*L. coriaria*	4	6	3	7	-		
*M. cucullatum*	6	5	1	1	8	-	
*H. brasiletto*	4	3	2	6	6	4	-

## Supplementary Materials

Supplementary materials can be found at http://www.mdpi.com/1422-0067/19/5/1286/s1.

## Abbreviations

LSC	Large single copy
SSC	Small single copy
IR	Inverted repeat
Cp	Chloroplast
ML	Maximum likelihood
BI	Bayesian inference
A	Adenine
T	Thymine
G	Guanine
C	Cytosine
