# Supplementary material for: Complete Chloroplast Genome of Cercis chuniana (Fabaceae) with Structural and Genetic Comparison to Six Species in Caesalpinioideae
Source: Int J Mol Sci. 2018 Apr 25;19(5):1286. doi: 10.3390/ijms19051286 (PMC5983592; doi:10.3390/ijms19051286)
Supplement: Supplementary file 1 [file ijms-19-01286-s001.pdf]

Supplementary Materials: Complete Chloroplast Genome of *Cercis chuniana* (Fabaceae) with Structural and Genetic Comparison to Six Species in Caesalpinioideae

Wanzhen Liu, Hanghui Kong, Juan Zhou, Peter W. Fritsch, Gang Hao and Wei Gong

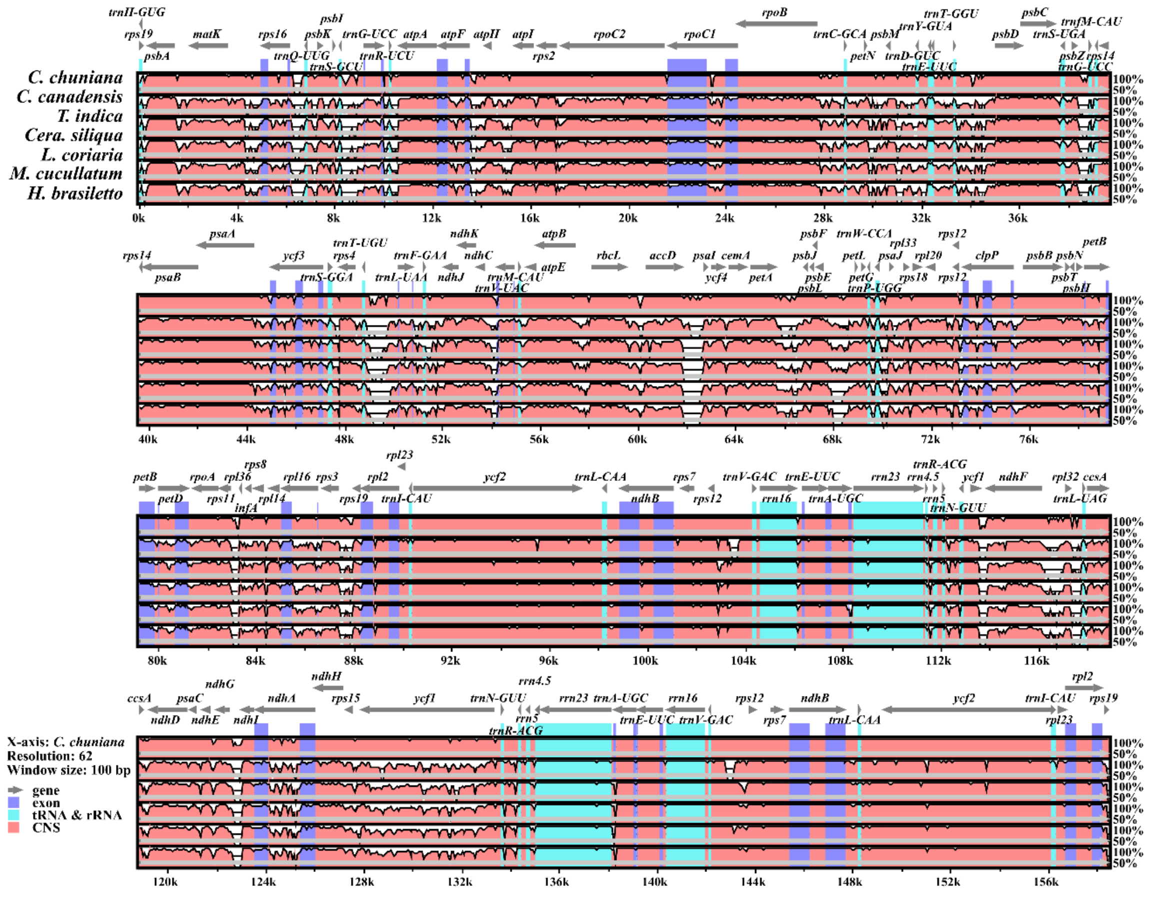

Figure S1. Structure comparison of the cp genomes in seven species of Fabaceae with mVISTA.

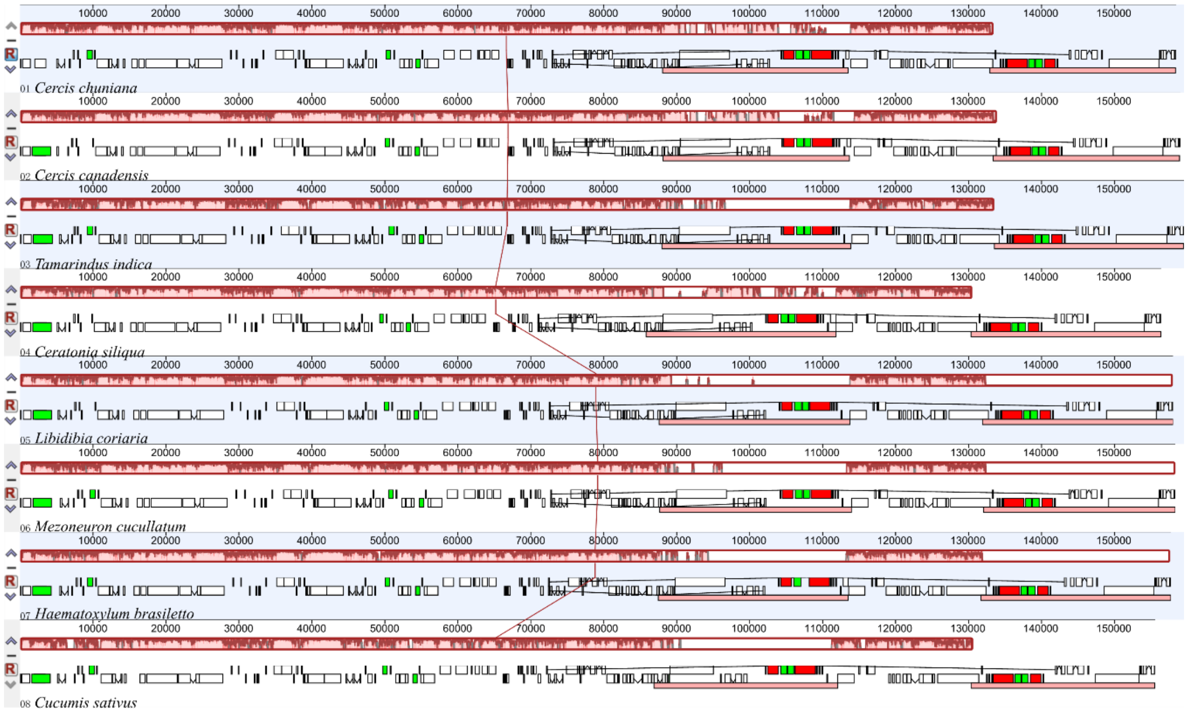

Figure S2. Mauve analysis for the cp genomes in seven species of Fabaceae.

Table S1. List of genes present in the *C. chuniana* cp genome.

Category	Gene Group	Gene Name			
Self-replication	rRNA genes	*rrn16*[a]	*rrn23*[a]	*rrn4.5*[a]	*rrn5*[a]
	tRNA genes	*trnA-UGC*[a,b]	*trnC-GCA*	*trnD-GUC*	*trnE-UUC*[a,b]
		trnF-GAA	*trnfM-CAU*	*trnG-UCC*[b]	*trnH-GUG*
		trnI-CAU[a]	*trnK-UUU*[b]	*trnL-CAA*[a]	*trnL-UAA*[b]
		trnL-UAG	*trnM-CAU*	*trnN-GUU*[a]	*trnP-UGG*
		trnQ-UUG	*trnR-ACG*[a]	*trnR-UCU*	*trnS-GCU*
		trnS-GGA	*trnS-UGA*	*trnT-GGU*	*trnT-UGU*
		trnV-GAC[a]	*trnV-UAC*[b]	*trnW-CCA*	*trnY-GUA*
	Small subunit of ribosome	*rps11*	*rps12*[a,d]	*rps14*	*rps15*
		rps16[b]	*rps18*	*rps19*[a]	*rps2*
		rps3	*rps4*	*rps7*[a]	*rps8*
	Large subunit of ribosome	*rpl2*[a,b]	*rpl14*	*rpl16*[b]	*rpl20*
		rpl23[a]	*rpl32*	*rpl33*	*rpl36*
	DNA-dependent RNA polymerase	*rpoA*	*rpoB*	*rpoC1*[b]	*rpoC2*
Genes for photosynthesis	Subunits of Photosystem I	*psaA*	*psaB*	*psaC*	*psaI*
		psaJ	*ycf3*[c]	*ycf4*	
	Subunits of Photosystem II	*psbA*	*psbB*	*psbC*	*psbD*
		psbE	*psbF*	*psbH*	*psbI*
		psbJ	*psbK*	*psbL*	*psbM*
		psbN	*psbT*	*psbZ*	
	Subunits of cytochrome	*petA*	*petB*[b]	*petD*[b]	*petG*
		petL	*petN*		
	Subunits of ATP synthase	*atpA*	*atpB*	*atpE*	*atpF*[b]
		atpH	*atpI*		
	Large subunit of Rubisco	*rbcL*			

	Subunits of NADH dehydrogenase	*ndhA*[b]	*ndhB*[a,b]	*ndhC*	*ndhD*
		ndhE	*ndhF*	*ndhG*	*ndhH*
		ndhI	*ndhJ*	*ndhK*	
Other genes	Maturase	*matK*			
	Envelope membrane protein	*cemA*			
	Subunit of acetyl-CoA	*accD*			
	C-type cytochrome Synthesis gene	*ccsA*			
	Protease	*clpP*[c]			
	Component of TIC complex	*ycf1*[a]			
	Function unknown	*ycf2*[a]			
	Translation initiation factor	*infA*			

a: Two gene copies in IR regions; b: gene containing a single intron; c: gene containing two introns; d: gene divided into two independent transcription units.

Table S2. Number and frequency of classified repeat types in the cp genomes of *C. chuniana* and related species.

Repeats	Compound SSR	A/T	C/G	AT/TA	TAA/TTA	TTAA	TTTA/AAAT	Total SSRs
C. chuniana	18 (19.8)	69 (94.5)	-	1 (1.37)	2 (2.74)	-	1 (1.37)	91
C. canadensis	20 (22.0)	67 (94.4)	1 (1.41)	3 (4.23)	-	-	-	91
T. indica	12 (14.1)	60 (82.2)	4 (5.48)	7 (9.59)	-	1 (1.37)	1 (1.37)	85
Cera. siliqua	13 (17.1)	61 (96.8)	-	2 (3.17)	-	-	-	76
L. coriaria	12 (18.2)	53 (98.2)	-	-	1 (1.85)	-	-	66
M. cucullatum	13 (16.5)	65 (98.5)	-	1 (1.52)	-	-	-	79
H. brasiletto	2 (5.26)	35 (97.2)	1 (2.78)	-	-	-	-	38
Average	13 (17.1)	59 (78.1)	1 (1.14)	2 (2.67)	0.43 (0.57)	0.14 (0.196%)	0.29 (0.391%)	75

Numbers in parentheses = each repeat type number/total repeat type number × 100.

Table S3. SSR primers of the *C. chuniana* cp genome designed by Primer3 (1.1.1-WINXP).

No.	SSR	DIR	F/R PRIMER1 (5′-3′)	Tm (°C)	PRO Size (bp)	Start (bp)	End (bp)
1	(A)12	F	CAGGATACAATCGCAATCAAACT	60.377	279	4301	4579
		R	TTTTTGGTTTTCGCCATTG	59.524			
2		F	TCAAAATGAACAGATCAGATCAAAA	59.997	175	4405	4579
		R	TTTTTGGTTTTCGCCATTGT	60.335			
3		F	TCAGGATACAATCGCAATCAA	59.126	272	4300	4571
		R	TTTCGCCATTGTAGTCTTTTCTT	59.341			
4	(T)12	F	ACAATGGCGAAAACCAAAAA	60.335	179	4560	4738
		R	TTACCCTCAATGAGTTGGGAA	59.415			
5		F	ACAATGGCGAAAACCAAAAA	60.335	181	4560	4740
		R	CATTACCCTCAATGAGTTGGG	59.298			
6		F	ACAATGGCGAAAACCAAAAA	60.335	177	4560	4736
		R	ACCCTCAATGAGTTGGGAAT	58.297			
7	(T)13	F	GAGCAAGAGAGAGGAAGGGG	60.468	201	4898	5098
		R	AAAAAGGCGCTCAACCTACA	59.883			
8		F	GAGCAAGAGAGAGGAAGGGG	60.468	210	4898	5107
		R	ATTTCCTTGAAAAAGGCGCT	60.204			
9		F	GAGCAAGAGAGAGGAAGGGG	60.468	200	4898	5097
		R	AAAAGGCGCTCAACCTACAG	59.517			
10	(T)12	F	CGTTGCTTTCTACCACATCG	59.342	207	6086	6292
		R	AATTTGAATTTTTGCCGAAAC	58.164			
11		F	ACGTTGCTTTCTACCACATCG	60.183	208	6085	6292
		R	AATTTGAATTTTTGCCGAAAC	58.164			
12		F	CGTTGCTTTCTACCACATCGT	60.183	207	6086	6292
		R	AATTTGAATTTTTGCCGAAAC	58.164			
13	(A)12	F	AAGTGACTCGATTTAGGGACGA	60.13	196	6582	6777
		R	CTTCCGTCCCAGAGCATATC	59.653			

14		F	AGTGACTCGATTTAGGGACGA	58.803	195	6583	6777
		R	CTTCCGTCCCAGAGCATATC	59.653			
15		F	CTCGATTTAGGGACGAATCA	57.765	190	6588	6777
		R	CTTCCGTCCCAGAGCATATC	59.653			
16	(T)11	F	TCGATTTCTCGAAAGCACTTC	59.583	223	7776	7998
		R	TTCTTCACGTCCCGGATTAC	59.933			
17		F	CGATTTCTCGAAAGCACTTCA	60.508	222	7777	7998
		R	TTCTTCACGTCCCGGATTAC	59.933			
18		F	TTTCGATTTCTCGAAAGCACT	59.101	225	7774	7998
		R	TTCTTCACGTCCCGGATTAC	59.933			
19	(A)10	F	TGCTTTTCCTAAGGATTCTTTCA	59.403	229	9615	9843
		R	CCGAACGTGAAACTTTGGTT	60.008			
20		F	TGCTTTTCCTAAGGATTCTTTCA	59.403	231	9615	9845
		R	AACCGAACGTGAAACTTTGG	60.008			
21		F	TGATTAAAGGATCCATGGCTAAA	59.824	267	9449	9715
		R	ACACCCCGCTTTTTAGATGA	59.569			
22	(T)10ctagaatcttataaaatcgtggatttttgatattaat tactaattattttattatatttttttattcctaattcttaagaatt aggaataaaaaatag(T)11	F	TGAATTGAACGAGAGACGCTT	60.008	280	10345	10624
		R	TGGAACTCTTTTTACTTCAGGAAC	58.082			
23		F	TGAATTGAACGAGAGACGCT	58.597	280	10345	10624
		R	TGGAACTCTTTTTACTTCAGGAAC	58.082			
24		F	TGAATTGAACGAGAGACGCTTA	60.018	280	10345	10624
		R	TGGAACTCTTTTTACTTCAGGAAC	58.082			
25	(A)11	F	CACCTGCCATTACCTCATCA	59.522	245	11963	12207
		R	TGGGGCGATGAAAGAAATAA	60.395			
26		F	CACCTGCCATTACCTCATCA	59.522	246	11963	12208
		R	TTGGGGCGATGAAAGAAATA	60.395			
27		F	TCATCTGCCCGAATGGTTAC	60.864	253	12094	12346
		R	ATTTTGAACAACAAAGGGCG	59.975			
28	(T)11	F	ACATAGGTCATCGATTCCGC	59.923	212	12891	13102

		R	AGACGCAGCGTGGTACTCTT	60.081			
29		F	ACATAGGTCATCGATTCCGC	59.923	218	12891	13108
		R	AAGTCTAGACGCAGCGTGGT	60.081			
30		F	TAGGTCATCGATTCCGCATT	60.435	209	12894	13102
		R	AGACGCAGCGTGGTACTCTT	60.081			
31	(T)12attcagaagtaattcgcgggatcatgcacctttat cctagttataacg(A)11	F	AAGAGTACCACGCTGCGTCT	60.081	270	13083	13352
		R	AAAGGGAGTGTGTGCGAGTT	59.769			
32		F	AAGAGTACCACGCTGCGTCT	60.081	271	13083	13353
		R	GAAAGGGAGTGTGTGCGAGT	60.307			
33		F	AAGAGTACCACGCTGCGTCT	60.081	268	13083	13350
		R	AGGGAGTGTGTGCGAGTTGT	60.779			
34	(TTA)5	F	TTTTTCATATGATCTCATCTCCTCTT	58.774	279	13479	13757
		R	TATGGGAAATCCTCCAAAAA	57.022			
35		F	TTTTCATATGATCTCATCTCCTCTT	57.539	278	13480	13757
		R	TATGGGAAATCCTCCAAAAA	57.022			
36		F	TTTTTCATATGATCTCATCTCCTCT	57.539	279	13479	13757
		R	TATGGGAAATCCTCCAAAAA	57.022			
37	(A)10	F	TGGAGGATTTCCCATAAAAATG	60.016	188	13742	13929
		R	GCCTTCTTCCTTCCCTTTTG	60.18			
38		F	TTTGGAGGATTTCCCATAAAAA	59.667	190	13740	13929
		R	GCCTTCTTCCTTCCCTTTTG	60.18			
39		F	TTGGAGGATTTCCCATAAAAA	58.412	189	13741	13929
		R	GCCTTCTTCCTTCCCTTTTG	60.18			
40	(A)10taaaatataaaa(AT)6	F	AAAAGCAAGAGCAGCAAAGC	59.911	242	14030	14271
		R	CAAGGTACTGCTGCAGGTCA	60.049			
41		F	AAAAGCAAGAGCAGCAAAGC	59.911	268	14030	14297
		R	TTGCTTCTATTGGACCTGGG	60.066			
42		F	AAAAGCAAGAGCAGCAAAGC	59.911	190	14030	14219
		R	CAGAGGGAAAAATACGGGGT	60.18			

43	(T)12	F	CCCCCACTCCTACCCTATTT	59.167	211	14838	15048
		R	ACGAATTTGGATTTCCCTCC	60.131			
44		F	CCCCCACTCCTACCCTATTT	59.167	206	14838	15043
		R	TTTGGATTTCCCTCCAATGA	60.244			
45		F	CCCCCACTCCTACCCTATTT	59.167	205	14838	15042
		R	TTGGATTTCCCTCCAATGAA	60.244			
46	(T)10	F	GGAGGGAAATCCAAATTCGT	60.131	246	15029	15274
		R	GTGTGTGCGGCTCAAGATAA	59.871			
47		F	TCATTGGAGGGAAATCCAAA	60.244	251	15024	15274
		R	GTGTGTGCGGCTCAAGATAA	59.871			
48		F	GGGAAATCCAAATTCGTCAA	59.739	243	15032	15274
		R	GTGTGTGCGGCTCAAGATAA	59.871			
49	(T)12caaattctttttatttg(A)10	F	ATGGCCAAAATGAACTCCTG	59.933	225	16934	17158
		R	AAAAATGAATGGCCTGATCG	59.901			
50		F	CATGGCCAAAATGAACTCCT	59.933	226	16933	17158
		R	AAAAATGAATGGCCTGATCG	59.901			
51		F	ACCATGGCCAAAATGAACTC	59.797	228	16931	17158
		R	AAAAATGAATGGCCTGATCG	59.901			
52	(T)12	F	AATTCCCCGAATTGGTTTTC	59.999	279	18934	19212
		R	ATATCCCGACACAGTGGCAT	60.226			
53		F	ACAGATCCTGGGGGAAAAGT	59.795	202	19011	19212
		R	ATATCCCGACACAGTGGCAT	60.226			
54		F	AACAGATCCTGGGGGAAAAG	60.298	203	19010	19212
		R	ATATCCCGACACAGTGGCAT	60.226			
55	(A)11	F	ACTTCGCCTCGATGGTTATG	60.096	178	23619	23796
		R	TATAGCGGTACCTCGCGAAA	60.73			
56		F	GGTTATGCCACGATGTCCTT	59.82	165	23632	23796
		R	TATAGCGGTACCTCGCGAAA	60.73			
57		F	ACTTCGCCTCGATGGTTATG	60.096	222	23619	23840

		R	GGGAATTGGTCTATGGTCGAT	60.032			
58	(T)10	F	CGTCGACCAATCCTTCCTAA	60.066	204	26880	27083
		R	GAATGTTTGCTACCCCGAAA	59.938			
59		F	CGTCGACCAATCCTTCCTAA	60.066	206	26880	27085
		R	GAGAATGTTTGCTACCCCGA	60.074			
60		F	CTCCGCCTACACAAGCAAAT	60.27	119	26965	27083
		R	GAATGTTTGCTACCCCGAAA	59.938			
61	(A)10	F	CCTAACTCCGGATATGGAATG	58.405	241	27795	28035
		R	TCATCGAACAGATCAAATCTCG	60.214			
62		F	ACCTAACTCCGGATATGGAATG	59.223	242	27794	28035
		R	TCATCGAACAGATCAAATCTCG	60.214			
63		F	TCCGGATATGGAATGTATGAAA	59.147	235	27801	28035
		R	TCATCGAACAGATCAAATCTCG	60.214			
64	(T)10	F	TCACGGATTCAATTGTTCAAA	59.002	210	28090	28299
		R	GAACGAAGGGCTCCAATACA	60.074			
65		F	TCACGGATTCAATTGTTCAAAA	60.335	210	28090	28299
		R	GAACGAAGGGCTCCAATACA	60.074			
66		F	TTCACGGATTCAATTGTTCAAA	60.335	211	28089	28299
		R	GAACGAAGGGCTCCAATACA	60.074			
67	(T)11	F	GAAGCACGATAATTTCCCGA	60.038	249	28367	28615
		R	CATGAACGATTCTTGGATTTGA	59.938			
68		F	GAAGCACGATAATTTCCCGA	60.038	250	28367	28616
		R	TCATGAACGATTCTTGGATTTG	59.938			
69		F	GAAGCACGATAATTTCCCGA	60.038	247	28367	28613
		R	TGAACGATTCTTGGATTTGAA	58.201			
70	(A)10	F	CGGAAGAATAGAATAAGAGTTGCC	59.682	280	30216	30495
		R	ATGATCGGACTGGAATCAGC	60.042			
71		F	TGATTGGAACCAATACAAATCAAA	60.44	239	30257	30495
		R	ATGATCGGACTGGAATCAGC	60.042			

72		F	TTGCCTTTTGATTATTTCAGATTG	59.537	261	30235	30495
		R	ATGATCGGACTGGAATCAGC	60.042			
73	(A)12	F	TTGGAATCCTGAATATGACGC	59.915	266	31470	31735
		R	CGATGGATACAAATCCACGA	59.35			
74		F	TGGAATCCTGAATATGACGCT	59.542	265	31471	31735
		R	CGATGGATACAAATCCACGA	59.35			
75		F	TGGAATCCTGAATATGACGC	58.521	265	31471	31735
		R	CGATGGATACAAATCCACGA	59.35			
76	(A)13	F	AATTGTTCATTCCAATCGCC	59.768	174	32637	32810
		R	TATGCCCACCGAATCTTAGC	60.06			
77		F	GCCCGACCACCATTCTACTA	59.955	280	32486	32765
		R	GTTTAACCCTAACCCCCTCG	59.698			
78		F	GCCCGACCACCATTCTACTA	59.955	252	32486	32737
		R	GGCAAACCAATCATTTCCTG	60.309			
79	(A)11	F	CCAATCAAAAGCCATTGGAA	60.807	244	33638	33881
		R	CGTGGAGTAAGGGAATAGCAA	59.234			
80		F	CATCTCCGCCAGATATTCCT	59.105	277	33605	33881
		R	CGTGGAGTAAGGGAATAGCAA	59.234			
81		F	CCAATCAAAAGCCATTGGAA	60.807	245	33638	33882
		R	ACGTGGAGTAAGGGAATAGCAA	60.021			
82	(T)12	F	TTGCTATTCCCTTACTCCACG	59.234	114	33861	33974
		R	TTGATCTTTTCTTGTGTTCGGA	59.724			
83		F	TTGCTATTCCCTTACTCCACG	59.234	103	33861	33963
		R	TTGTGTTCGGAAATCAAAGAAA	59.603			
84		F	TGCTATTCCCTTACTCCACG	57.85	113	33862	33974
		R	TTGATCTTTTCTTGTGTTCGGA	59.724			
85	(A)12	F	TCTGAAAGAAAGGAAAATAGAACAAA	58.983	268	34397	34664
		R	TGAATCTTGTTCGTGACATTGA	59.174			
86		F	TCTGAAAGAAAGGAAAATAGAACAAAA	60.05	268	34397	34664

		R	TGAATCTTGTTCGTGACATTGA	59.174			
87		F	TCTGAAAGAAAGGAAAATAGAACAA	57.818	268	34397	34664
		R	TGAATCTTGTTCGTGACATTGA	59.174			
88	(A)10	F	GCATGCCCTTTTGTGAAGTT	60.118	121	37948	38068
		R	AATGCATCATGGGGGTTTTA	60.016			
89		F	AGCATGCCCTTTTGTGAAGT	59.74	122	37947	38068
		R	AATGCATCATGGGGGTTTTA	60.016			
90		F	GCATGCCCTTTTGTGAAGTT	60.118	111	37948	38058
		R	GGGGGTTTTACCATGAATCC	60.245			
91	(A)14taataattctaattaatttctagttaaaaaaatcaa acctcaa(T)11	F	TCTTTGCCAAGGAGAAGACG	60.508	250	38858	39107
		R	TTGAGGTCACGGGTTCAAAT	60.353			
92		F	TCTTTGCCAAGGAGAAGACG	60.508	273	38858	39130
		R	GGTAGCTCGCAAGGCTCATA	60.511			
93		F	TCTTTGCCAAGGAGAAGACG	60.508	249	38858	39106
		R	TGAGGTCACGGGTTCAAATC	60.903			
94	(T)12	F	GAGTTTCCTGCCTCATACGG	59.694	266	45175	45440
		R	GAGTGGAAATGCCAATCCTT	58.984			
95		F	GAGTTTCCTGCCTCATACGG	59.694	267	45175	45441
		R	AGAGTGGAAATGCCAATCCTT	59.954			
96		F	GCCTCATACGGCTCAGAAGT	59.459	257	45184	45440
		R	GAGTGGAAATGCCAATCCTT	58.984			
97	(T)11	F	ATCGGCTCAAAACCTCGTTA	59.708	275	45747	46021
		R	TATGGCCGTGATCTGTCATT	58.953			
98		F	TATCGGCTCAAAACCTCGTT	59.708	276	45746	46021
		R	TATGGCCGTGATCTGTCATT	58.953			
99		F	ATCGGCTCAAAACCTCGTTA	59.708	274	45747	46020
		R	ATGGCCGTGATCTGTCATTA	58.953			
100	(A)10gaatcgaccgttcgagtattcaaaattgcatgata aaaatgatataaagagggacata(AT)8	F	GAAAGAGAAAGGCCCAATCC	60.017	225	49794	50018
		R	TTGGTCCAATCCGAAATGAT	60.133			

101		F	GAAAGAGAAAGGCCCAATCC	60.017	231	49794	50024
		R	CCATATTTGGTCCAATCCGA	60.523			
102		F	ATAGGGAGGGTCTGTCTGCC	60.477	279	49740	50018
		R	TTGGTCCAATCCGAAATGAT	60.133			
103	(A)13	F	CAACCGTTTCGGCATAATTT	59.832	217	50110	50326
		R	GGAAAACAGGATTTGGCTCA	60.051			
104		F	CAACCGTTTCGGCATAATTT	59.832	141	50110	50250
		R	GGTTTCCATACCAAGGCTCA	59.933			
105		F	AATGAATTCAACCGTTTCGG	59.801	225	50102	50326
		R	GGAAAACAGGATTTGGCTCA	60.051			
106	(T)10	F	CGGCCATAAACTGGAGAGTC	59.694	274	53208	53481
		R	ATCCATGGGCAATGAGTTTC	59.756			
107		F	CCATAGAGAAGCGGCCATAA	60.188	199	53197	53395
		R	GTTTATGCATGGCGAAAAGG	60.456			
108		F	GTACCATAGAGAAGCGGCCA	60.235	202	53194	53395
		R	GTTTATGCATGGCGAAAAGG	60.456			
109	(T)10caagcgcggaaacctcaggaccagaagcggta ggatttattctcataataaaatatatcaa(T)10	F	CCTGGGGGAAAAGCTACATC	60.817	256	57214	57469
		R	GAATTGGATTGGTTGGATGG	59.991			
110		F	TATTTGAGCGATACGTCCCA	59.136	221	57249	57469
		R	GAATTGGATTGGTTGGATGG	59.991			
111		F	CGGACCAATTATTTGAGCGA	60.961	230	57240	57469
		R	GAATTGGATTGGTTGGATGG	59.991			
112	(A)11	F	TCATTGGCCCTTATTTCAGC	60.038	235	57665	57899
		R	TTCATATGTATGGCGCAACC	59.405			
113		F	TTTTCGTGGGCTAATTCTGC	60.209	166	57734	57899
		R	TTCATATGTATGGCGCAACC	59.405			
114		F	TCATTGGCCCTTATTTCAGC	60.038	278	57665	57942
		R	CCATGGTATTTGATTTGCCA	59.232			
115	(A)10	F	CTGCAGAAAGAATCATGTGGA	58.862	278	61962	62239

		R	AATAGGGATTCGGGTTCTCG	60.278			
116		F	TGCAGAAAGAATCATGTGGA	57.743	277	61963	62239
		R	AATAGGGATTCGGGTTCTCG	60.278			
117		F	ACTGCAGAAAGAATCATGTGGA	59.74	279	61961	62239
		R	AATAGGGATTCGGGTTCTCG	60.278			
118		F	GCGAAATTAGGCAAGAGGAA	59.433	183	62057	62239
		R	AATAGGGATTCGGGTTCTCG	60.278			
119		F	GCGAAATTAGGCAAGAGGAA	59.433	176	62057	62232
		R	ATTCGGGTTCTCGGGAGTAG	60.455			
120		F	AAGCGAATTCTTTCTTCCGC	60.821	201	62039	62239
		R	AATAGGGATTCGGGTTCTCG	60.278			
121	(T)10	F	CGAGAACCCGAATCCCTATT	60.278	180	62220	62399
		R	TGATTCTTGTCTGATAATTTCCATTT	59.349			
122		F	CGAGAACCCGAATCCCTATT	60.278	182	62220	62401
		R	TTTGATTCTTGTCTGATAATTTCCAT	59.349			
123		F	CGAGAACCCGAATCCCTATT	60.278	181	62220	62400
		R	TTGATTCTTGTCTGATAATTTCCATT	59.349			
124		F	CAATTGCAATGGCTTCCTTA	58.769	261	62762	63022
		R	ATACGTTCTGATCGCCAACC	59.962			
125		F	CAATTGCAATGGCTTCCTTA	58.769	250	62762	63011
		R	TCGCCAACCCATACTAGACC	59.955			
126		F	TTGTCCCTTTAGTGGGCCT	59.536	280	62732	63011
		R	TCGCCAACCCATACTAGACC	59.955			
127	(A)11tgaacttgaagaataaaataaatatgaatgctttctt(A)10	F	GAGAATTTGACCCCACGAGA	60.05	241	63474	63714
		R	CACCTTCACCTCATTCGTCA	59.676			
128		F	GAATTTGACCCCACGAGAAA	59.91	239	63476	63714
		R	CACCTTCACCTCATTCGTCA	59.676			
129		F	ATTGGCCTATTTCTTGCGTG	60.096	200	63515	63714
		R	CACCTTCACCTCATTCGTCA	59.676			

130	(T)10	F	AGAGCCCCTTTTCCTTGTGT	60.11	257	66012	66268
		R	TTTGCTCAATAAAATTCCCTCTC	58.809			
131		F	CCTAGAGCCCCTTTTCCTTG	60.193	260	66009	66268
		R	TTTGCTCAATAAAATTCCCTCTC	58.809			
132		F	AGAGCCCCTTTTCCTTGTGT	60.11	255	66012	66266
		R	TGCTCAATAAAATTCCCTCTCTTT	59.663			
133	(AAAT)5	F	GAGAGGGAATTTTATTGAGCAAA	58.809	229	66246	66474
		R	ACTTTTTCTGGGTGACGGTG	60.005			
134		F	AGAGAGGGAATTTTATTGAGCAAA	59.663	230	66245	66474
		R	ACTTTTTCTGGGTGACGGTG	60.005			
135		F	AAAGAGAGGGAATTTTATTGAGCA	59.663	232	66243	66474
		R	ACTTTTTCTGGGTGACGGTG	60.005			
136	(T)12	F	GCGAGTAAAATGTGAATCCGA	60.089	269	67896	68164
		R	CGCATCGCGACTATTGATT	59.807			
137		F	GGATCGCGAGTAAAATGTGAA	60.089	274	67891	68164
		R	CGCATCGCGACTATTGATT	59.807			
138		F	TCGCGAGTAAAATGTGAATCC	60.089	271	67894	68164
		R	CGCATCGCGACTATTGATT	59.807			
139	(AT)6	F	ACCTACGACATCGGGTTTTG	59.853	279	69425	69703
		R	CAAGGGATCAATTGAGATCG	58.089			
140		F	ACCTACGACATCGGGTTTTG	59.853	280	69425	69704
		R	ACAAGGGATCAATTGAGATCG	58.995			
141		F	TGTGACCCCCAAAAATAAAA	57.847	258	69502	69759
		R	CGGGTTCAAATCCTGTCATC	60.317			
142	(T)11gtt(A)13	F	ATTTTTAGGGAATGCTCGGG	60.272	274	70003	70276
		R	TCGCATTTAAAATCCTCCTTC	58.325			
143		F	ATTTTTAGGGAATGCTCGGG	60.272	274	70003	70276
		R	TCGCATTTAAAATCCTCCTTCT	59.251			
144		F	ATTTTTAGGGAATGCTCGGG	60.272	276	70003	70278

		R	TCTCGCATTTAAAATCCTCCTT	59.251			
145	(T)11	F	GGATGCCTTGATATTCCCCT	60.117	277	70372	70648
		R	TAGGATATTGTTGCCCCACC	59.645			
146		F	GGATGCCTTGATATTCCCCT	60.117	274	70372	70645
		R	GATATTGTTGCCCCACCCTA	59.645			
147		F	GGATGCCTTGATATTCCCCT	60.117	254	70372	70625
		R	GCCATTACATTTCCAGGCTC	59.533			
148	(A)10	F	AAAGCGAGTCGACCACTAGAA	59.135	224	71486	71709
		R	CATCAAAATGAGCAACCACG	60.111			
149		F	AAAAGCGAGTCGACCACTAGA	59.135	225	71485	71709
		R	CATCAAAATGAGCAACCACG	60.111			
150		F	AAAGCGAGTCGACCACTAGAAC	59.95	224	71486	71709
		R	CATCAAAATGAGCAACCACG	60.111			
151	(T)14caaatcaaatacaaatatataataaaaaagaaaa tcttg(T)10	F	CTATGATGGTTCCGCTGCTT	60.235	239	73743	73981
		R	TGGGAAAAGGGCTATTTACG	59.075			
152		F	ACTATGATGGTTCCGCTGCT	59.723	240	73742	73981
		R	TGGGAAAAGGGCTATTTACG	59.075			
153		F	CTATGATGGTTCCGCTGCTT	60.235	240	73743	73982
		R	CTGGGAAAAGGGCTATTTACG	59.971			
154	(T)10	F	CGTAAATAGCCCTTTTCCCA	59.075	200	73962	74161
		R	TCTAGCATTCCCTCACGCTT	59.978			
155		F	CGTAAATAGCCCTTTTCCCA	59.075	266	73962	74227
		R	AGTATGCATGGGATTAGCCG	59.945			
156		F	CGTAAATAGCCCTTTTCCCAG	59.971	200	73962	74161
		R	TCTAGCATTCCCTCACGCTT	59.978			
157	(T)10cttacatttttcaaaaaagacgacttttgacccgttt acttatattataagtattataag(A)11	F	TGCATACGGTTCACCAAAAA	59.969	263	74482	74744
		R	ACGATTTGGATTCGATCTGG	59.894			
158		F	ATAGCGGGCTTTTTCGAACT	60.224	185	74560	74744
		R	ACGATTTGGATTCGATCTGG	59.894			

159		F	GATGCATACGGTTCACCAAA	59.4	265	74480	74744
		R	ACGATTTGGATTCGATCTGG	59.894			
160	(A)10	F	ACTTTGATGCGGAAGCGTAA	60.772	264	75338	75601
		R	ATACGCAATGGTGTAGGGGA	60.214			
161		F	CTTTGATGCGGAAGCGTAAC	60.772	263	75339	75601
		R	ATACGCAATGGTGTAGGGGA	60.214			
162		F	ACTTTGATGCGGAAGCGTAA	60.772	265	75338	75602
		R	AATACGCAATGGTGTAGGGG	59.708			
163	(A)11t(A)11	F	ATATTTAGACCTCGCAGCCG	59.343	251	78432	78682
		R	AGATTCATACCGGAACCACG	59.813			
164		F	ATATTTAGACCTCGCAGCCG	59.343	246	78432	78677
		R	CATACCGGAACCACGATGAT	60.603			
165		F	ATATTTAGACCTCGCAGCCG	59.343	250	78432	78681
		R	GATTCATACCGGAACCACGA	60.723			
166	(T)10	F	TTCGAGTATTGGGATTTGTGC	59.952	271	81307	81577
		R	CCAATTAGAATTGCCTCCCC	60.633			
167		F	TCGAGTATTGGGATTTGTGCT	59.583	270	81308	81577
		R	CCAATTAGAATTGCCTCCCC	60.633			
168		F	TCGAGTATTGGGATTTGTGC	58.574	270	81308	81577
		R	CCAATTAGAATTGCCTCCCC	60.633			
169	(A)13	F	GAACGGAGGCCCTTATTTTC	59.909	206	83360	83565
		R	TTTTGATTCAACCAGAGGGC	60.051			
170		F	CTACGGATCAGCCGACATTT	60.096	240	83326	83565
		R	TTTTGATTCAACCAGAGGGC	60.051			
171		F	ACTATAATTCGTCCCCGCCT	59.82	258	83308	83565
		R	TTTTGATTCAACCAGAGGGC	60.051			
172	(T)10	F	GATTGTTGCCCGTGACTTTT	59.978	269	84299	84567
		R	GCCCGAGAATTGAGACAGTT	59.288			
173		F	GATTGTTGCCCGTGACTTTT	59.978	234	84299	84532

		R	AGTTTCATTAGCACCCGAGG	59.195			
174		F	ATGATTGTTGCCCGTGACTT	60.384	271	84297	84567
		R	GCCCGAGAATTGAGACAGTT	59.288			
175	(T)10atctgttctttcagtgcaaagcaaaggacgaagt(A)10	F	CCGCTGTTATCGGCTACATT	60.117	240	84830	85069
		R	ATAGCAGCATCCAAAATGCC	60.067			
176		F	CCGCTGTTATCGGCTACATT	60.117	237	84830	85066
		R	GCAGCATCCAAAATGCCTAT	60.067			
177		F	CGGCTACATTCAAATGGGTT	59.823	230	84840	85069
		R	ATAGCAGCATCCAAAATGCC	60.067			
178	(T)11	F	TTATTGACTCGGGGCTCTTG	60.206	176	86061	86236
		R	TGAAAGGGTGAAAGAAAGAGAGA	59.508			
179		F	TTCCCATCGCTTCTCGATTA	60.681	270	85967	86236
		R	TGAAAGGGTGAAAGAAAGAGAGA	59.508			
180		F	TATTGACTCGGGGCTCTTGA	60.734	175	86062	86236
		R	TGAAAGGGTGAAAGAAAGAGAGA	59.508			
181		F	TCTCTCTTTCTTTCACCCTTTCA	59.508	185	86214	86398
		R	CAACTCATCGCTTCGCATTA	59.976			
182		F	TCTCTCTTTCTTTCACCCTTTCA	59.508	181	86214	86394
		R	TCATCGCTTCGCATTATCTG	59.937			
183		F	TCTCTCTTTCTTTCACCCTTTCA	59.508	179	86214	86392
		R	ATCGCTTCGCATTATCTGGA	60.711			
184	(T)13	F	TTTTGTCCCATAATTCCCCA	59.988	261	87309	87569
		R	TGAGTCAAAAGCTGAAGGTCC	59.463			
185		F	TTTGTCCCATAATTCCCCAC	59.481	260	87310	87569
		R	TGAGTCAAAAGCTGAAGGTCC	59.463			
186		F	TTTTGTCCCATAATTCCCCA	59.988	267	87309	87575
		R	GCAAGTTGAGTCAAAAGCTGAA	59.677			
187	(T)10	F	TTTTCACCTCATACGGCTCC	60.074	194	102761	102954
		R	CGCTCAATGAGAAAATGGGT	60.074			

188		F	CATACGGCTCCTCGTTCAAT	60.096	185	102770	102954
		R	CGCTCAATGAGAAAATGGGT	60.074			
189		F	TTGGGGGTGATAGTGGAAAA	60.162	264	102691	102954
		R	CGCTCAATGAGAAAATGGGT	60.074			
190	(T)11	F	TCCGATTCATGAGGATACGA	59.027	205	114447	114651
		R	GGGGTAAAGAAGGGCAAAAA	60.285			
191		F	GTCCGATTCATGAGGATACGA	59.909	206	114446	114651
		R	GGGGTAAAGAAGGGCAAAAA	60.285			
192		F	CCGATTCATGAGGATACGAAA	59.908	204	114448	114651
		R	GGGGTAAAGAAGGGCAAAAA	60.285			
193	(T)10	F	GGAACTGGAAGTGGGATGAA	59.903	231	116031	116261
		R	TGAAGTATTGGGCAAAGCAA	59.301			
194		F	GGAACTGGAAGTGGGATGAA	59.903	232	116031	116262
		R	TTGAAGTATTGGGCAAAGCA	59.301			
195		F	GTTGCCGTCGGAAAAAGTAG	59.747	274	115989	116262
		R	TTGAAGTATTGGGCAAAGCA	59.301			
196	(A)11	F	GGCAGTACGCCGAAAATAGA	60.23	273	116865	117137
		R	TTGGAACTGCCATTCAAAAA	59.133			
197		F	AAGGCAGTACGCCGAAAATA	59.742	275	116863	117137
		R	TTGGAACTGCCATTCAAAAA	59.133			
198		F	ATAAAGGCAGTACGCCGAAA	59.742	278	116860	117137
		R	TTGGAACTGCCATTCAAAAA	59.133			
199		F	GCGAAATCTCTTTATACGGGG	59.943	262	117227	117488
		R	CAGAAGAACGAAATGGGTTCA	60.096			
200		F	TGAATTAGACGAATGCGAAGAA	59.853	194	117295	117488
		R	CAGAAGAACGAAATGGGTTCA	60.096			
201		F	GCGAAATCTCTTTATACGGGG	59.943	261	117227	117487
		R	AGAAGAACGAAATGGGTTCAA	58.682			
202		F	CGGGATGGGGATTCTTATTT	59.978	205	117589	117793

		R	ACCATAGCGGCTTGCTCTAA	60.003			
203		F	GGGGATTCTTATTTTCCCCA	59.957	199	117595	117793
		R	ACCATAGCGGCTTGCTCTAA	60.003			
204		F	CGGGATGGGGATTCTTATTT	59.978	228	117589	117816
		R	AGAGCAGCGTGTCTACCGAT	60.043			
205	(T)10	F	TCAATTCCCGATTTCCATTC	59.699	208	117898	118105
		R	TCCTTTTTCGGACGAATCAT	59.505			
206		F	AATTCCCGATTTCCATTCCT	59.605	206	117900	118105
		R	TCCTTTTTCGGACGAATCAT	59.505			
207		F	AGAGCATCTCGGTTCGAGTC	59.56	275	117831	118105
		R	TCCTTTTTCGGACGAATCAT	59.505			
208	(A)13	F	TGGGATCCAAAGGAAACTTG	59.903	223	118733	118955
		R	CAAGCCCCTTACTCACTCCA	60.246			
209		F	TGAGGCATGGGGATCATATT	60.118	248	118708	118955
		R	CAAGCCCCTTACTCACTCCA	60.246			
210		F	GAGGCATGGGGATCATATTG	60.118	247	118709	118955
		R	CAAGCCCCTTACTCACTCCA	60.246			
211	(T)10	F	TCTCGCAAACACCAACCATA	60.111	209	119054	119262
		R	TCAGTTGACAAGGTCGAAGC	59.01			
212		F	AAAACTGCGCATAAACCGTC	60.138	269	118994	119262
		R	TCAGTTGACAAGGTCGAAGC	59.01			
213		F	GAAAACTGCGCATAAACCGT	60.138	270	118993	119262
		R	TCAGTTGACAAGGTCGAAGC	59.01			
214	(T)10gtctattttgagcacgcgtttttgtcagtaaaaaaa acgtattcgt(G)10	F	CAGAAAAACGCGTGTTCAAA	59.888	269	120742	121010
		R	GATACCTTGGGACGGCTGTA	59.955			
215		F	CAGAAAAACGCGTGTTCAAA	59.888	178	120742	120919
		R	CCGACGGATTTCTTGAGTGT	60.111			
216		F	CAGAAAAACGCGTGTTCAAA	59.888	237	120742	120978
		R	TTGCTTCTGCTCCAAGAACA	59.716			

217	(A)11	F	CGAGCATCATTGATCAACTCC	60.619	243	121662	121904
		R	GCAACAGCAATAGCAATCCA	59.839			
218		F	CGAGCATCATTGATCAACTCC	60.619	273	121662	121934
		R	TGGCTCGCCAGTAAGAAATC	60.352			
219		F	CGAGCATCATTGATCAACTCC	60.619	274	121662	121935
		R	GTGGCTCGCCAGTAAGAAAT	59.34			
220	(A)10	F	CCAACGAAAAGGCAGAAAAA	60.215	179	122334	122512
		R	TCCTGGTCCTATCAATAAGGTCAT	60.095			
221		F	TAGTCCCAACGAAAAGGCAG	60.241	184	122329	122512
		R	TCCTGGTCCTATCAATAAGGTCAT	60.095			
222		F	CTAGTCCCAACGAAAAGGCA	60.241	185	122328	122512
		R	TCCTGGTCCTATCAATAAGGTCAT	60.095			
223	(TAA)5	F	GCTCCTCGAGGCCATAAATA	59.277	209	124144	124352
		R	AAGGGATGAGAGCAATTCAGA	58.875			
224		F	GCTCCTCGAGGCCATAAATA	59.277	207	124144	124350
		R	GGGATGAGAGCAATTCAGAAG	58.875			
225		F	GCTCCTCGAGGCCATAAATA	59.277	208	124144	124351
		R	AGGGATGAGAGCAATTCAGAAG	59.848			
226	(T)11cgttcctattcttctttttgtttttatgtgcataaagg gcac(T)10(A)14	F	ACGGGATCCCTCTTTCTTTG	60.434	207	124509	124715
		R	CAGGCAGTACTCCCCACAAT	59.989			
227		F	CGGGATCCCTCTTTCTTTGT	60.434	206	124510	124715
		R	CAGGCAGTACTCCCCACAAT	59.989			
228		F	GACGGGATCCCTCTTTCTTT	59.513	208	124508	124715
		R	CAGGCAGTACTCCCCACAAT	59.989			
229	(T)11	F	TGATACTCATTCATTTTGAATTTGG	59.306	262	127579	127840
		R	TGCACACACGCATTGTCTTA	59.902			
230		F	TGATACTCATTCATTTTGAATTTGG	59.306	263	127579	127841
		R	ATGCACACACGCATTGTCTT	60.192			
231		F	TGATACTCATTCATTTTGAATTTGGT	60.016	262	127579	127840

		R	TGCACACACGCATTGTCTTA	59.902			
232	(T)13	F	GGTATCGACCCAGTCCTCAA	59.927	146	129101	129246
		R	TGACGGATGATATCTCTATCAAGAA	59.157			
233		F	GGTATCGACCCAGTCCTCAA	59.927	162	129101	129262
		R	CAAAAACTATTTGATATGACGGATG	58.909			
234		F	GGTATCGACCCAGTCCTCAA	59.927	163	129101	129263
		R	ACAAAAACTATTTGATATGACGGATG	59.617			
235	(T)10	F	TGAATTCTTTCTTCATTTGCTTCA	60.232	169	129912	130080
		R	TCCGGAAATCGCATTATTTT	59.393			
236		F	TGAATTCTTTCTTCATTTGCTTCA	60.232	175	129912	130086
		R	GATTAGTCCGGAAATCGCAT	59.001			
237		F	TGAATTCTTTCTTCATTTGCTTCA	60.232	168	129912	130079
		R	CCGGAAATCGCATTATTTTT	58.937			
238	(T)11	F	TGATGATCCATGCTGCTGTT	60.233	270	130459	130728
		R	CGGAAAAGGATCCAATGAAA	59.872			
239		F	TTGATGATCCATGCTGCTGT	60.233	271	130458	130728
		R	CGGAAAAGGATCCAATGAAA	59.872			
240		F	TTGATGATCCATGCTGCTGT	60.233	221	130458	130678
		R	TTAACAATTTGGGGAACGGA	60.16			
241	(T)10	F	TTCCGTTCCCCAAATTGTTA	60.16	112	130658	130769
		R	TTTTCGTCTGAAACCTTGGC	60.227			
242		F	CCGTTCCCCAAATTGTTAAA	59.671	110	130660	130769
		R	TTTTCGTCTGAAACCTTGGC	60.227			
243		F	TTCCGTTCCCCAAATTGTTA	60.16	258	130658	130915
		R	TTTCAAGTTCCCGAATGGTT	59.406			
244	(T)11	F	GTCCGTTCAAGAAAGGATCG	59.67	254	131977	132230
		R	TTTTGTGGAAATGATGCAAAA	59.039			
245		F	GTCCGTTCAAGAAAGGATCG	59.67	259	131977	132235
		R	TCAACTTTTGTGGAAATGATGC	59.982			

246		F	GTCCGTTCAAGAAAGGATCG	59.67	255	131977	132231
		R	CTTTTGTGGAAATGATGCAAAA	59.979			
247	(A)10	F	CGCTCAATGAGAAAATGGGT	60.074	194	143541	143734
		R	TTTTCACCTCATACGGCTCC	60.074			
248		F	CGCTCAATGAGAAAATGGGT	60.074	185	143541	143725
		R	CATACGGCTCCTCGTTCAAT	60.096			
249		F	CGCTCAATGAGAAAATGGGT	60.074	264	143541	143804
		R	TTGGGGGTGATAGTGGAAAA	60.162			

Table S4. Species included in phylogenetic analysis. (* = outgroup; # = species in compared analysis.)

Code	Accession	Organism	Taxonomy
1 #	KJ468095	*Libidibia coriaria* (Jacq.) Schltdl.	Fabaceae; Caesalpinioideae; Caesalpinia clade
2 #	KU569489	*Mezoneuron cucullatum* (Roxb.) Wight & Arn.	Fabaceae; Caesalpinioideae; Caesalpinia clade
3 #	KJ468097	*Haematoxylum brasiletto* H.Karst.	Fabaceae; Caesalpinioideae; Cassia clade
4	NC_030193	*Senna tora (L.)* Roxb.	Fabaceae; Caesalpinioideae; Cassia clade
5	NC_034985	*Acacia dealbata* Link	Fabaceae; Caesalpinioideae; mimosoid clade; Acacieae
6	NC_026134	*Acacia ligulata* Benth.	Fabaceae; Caesalpinioideae; mimosoid clade; Acacieae
7	LN885332	*Acacia yorkrakinensis* C.A.Gardner subsp. *acrita* R.S.Cowan & Maslin	Fabaceae; Caesalpinioideae; mimosoid clade; Acacieae
8	LN885330	*Acacia xanthina* Benth.	Fabaceae; Caesalpinioideae; mimosoid clade; Acacieae
9	LN885328	*Acacia woodmaniorum* Maslin & Buscumb	Fabaceae; Caesalpinioideae; mimosoid clade; Acacieae
10	LN885327	*Acacia websteri* Maiden & Blakeley	Fabaceae; Caesalpinioideae; mimosoid clade; Acacieae
11	LN885326	*Acacia uncinella Benth.*	Fabaceae; Caesalpinioideae; mimosoid clade; Acacieae
12	LN885325	*Acacia umbraculiformis* Maslin & Buscumb	Fabaceae; Caesalpinioideae; mimosoid clade; Acacieae
13	LN885324	*Acacia tysonii* Luehm.	Fabaceae; Caesalpinioideae; mimosoid clade; Acacieae
14	LN885323	*Acacia tetragonophylla* F.Muell.	Fabaceae; Caesalpinioideae; mimosoid clade; Acacieae
15	LN885319	*Acacia sulcaticaulis* Maslin & Buscumb	Fabaceae; Caesalpinioideae; mimosoid clade; Acacieae
16	LN885318	*Acacia stereophylla* Meissner var. *stereophylla*	Fabaceae; Caesalpinioideae; mimosoid clade; Acacieae
17	LN885317	*Acacia stanleyi* Maslin	Fabaceae; Caesalpinioideae; mimosoid clade; Acacieae
18	LN885316	*Acacia sibina* Maslin	Fabaceae; Caesalpinioideae; mimosoid clade; Acacieae

19	LN885315	*Acacia sclerosperma* F.Muell. subsp. *sclerosperma*	Fabaceae; Caesalpinioideae; mimosoid clade; Acacieae
20	LN885313	*Acacia scleroclada* Maslin	Fabaceae; Caesalpinioideae; mimosoid clade; Acacieae
21	LN885312	*Acacia scirpifolia* Meissner	Fabaceae; Caesalpinioideae; mimosoid clade; Acacieae
22	LN885311	*Acacia scalena* Maslin	Fabaceae; Caesalpinioideae; mimosoid clade; Acacieae
23	LN885310	*Acacia rostellifera* Benth.	Fabaceae; Caesalpinioideae; mimosoid clade; Acacieae
24	LN885308	*Acacia restiacea* Benth.	Fabaceae; Caesalpinioideae; mimosoid clade; Acacieae
25	LN885306	*Acacia resinosa* R.S.Cowan & Maslin	Fabaceae; Caesalpinioideae; mimosoid clade; Acacieae
26	LN885304	*Acacia resinimarginea* W.Fitzg.	Fabaceae; Caesalpinioideae; mimosoid clade; Acacieae
27	LN885301	*Acacia ramulosa* W.Fitzg. var. *ramulosa*	Fabaceae; Caesalpinioideae; mimosoid clade; Acacieae
28	LN885300	*Acacia puncticulata* Maslin	Fabaceae; Caesalpinioideae; mimosoid clade; Acacieae
29	LN885299	*Acacia prainii* Maiden	Fabaceae; Caesalpinioideae; mimosoid clade; Acacieae
30	LN885298	*Acacia oldfieldii* F.Muell.	Fabaceae; Caesalpinioideae; mimosoid clade; Acacieae
31	LN885296	*Acacia obtecta* Maiden & Blakeley	Fabaceae; Caesalpinioideae; mimosoid clade; Acacieae
32	LN885295	*Acacia neurophylla* W.Fitzg. subsp. *erugata* R.S.Cowan & Maslin	Fabaceae; Caesalpinioideae; mimosoid clade; Acacieae
33	LN885293	*Acacia murrayana* Benth.	Fabaceae; Caesalpinioideae; mimosoid clade; Acacieae
34	LN885291	*Acacia merrallii* F.Muell.	Fabaceae; Caesalpinioideae; mimosoid clade; Acacieae
35	LN885289	*Acacia longispinea* Morrison	Fabaceae; Caesalpinioideae; mimosoid clade; Acacieae
36	LN885287	*Acacia longiphyllodinea* Maiden	Fabaceae; Caesalpinioideae; mimosoid clade; Acacieae
37	LN885285	*Acacia lineolata* Benth. subsp. *lineolata*	Fabaceae; Caesalpinioideae; mimosoid clade; Acacieae
38	LN885284	*Acacia lasiocalyx* C.R.P.Andrews	Fabaceae; Caesalpinioideae; mimosoid clade; Acacieae
39	LN885282	*Acacia kochii* Ewart & Jean White	Fabaceae; Caesalpinioideae; mimosoid clade; Acacieae
40	LN885281	*Acacia karina* Maslin & Buscumb	Fabaceae; Caesalpinioideae; mimosoid clade; Acacieae
41	LN885279	*Acacia jibberdingensis* Maiden & Blakeley	Fabaceae; Caesalpinioideae; mimosoid clade; Acacieae
42	LN885277	*Acacia jennerae* Maiden	Fabaceae; Caesalpinioideae; mimosoid clade; Acacieae
43	LN885276	*Acacia inceana* Domin subsp. *conformis* R.S.Cowan & Maslin	Fabaceae; Caesalpinioideae; mimosoid clade; Acacieae
44	LN885274	*Acacia heteroclita* Meissner subsp. *heteroclita*	Fabaceae; Caesalpinioideae; mimosoid clade; Acacieae
45	LN885273	*Acacia hemiteles* Benth.	Fabaceae; Caesalpinioideae; mimosoid clade; Acacieae
46	LN885271	*Acacia gibbosa* R.S.Cowan & Maslin	Fabaceae; Caesalpinioideae; mimosoid clade; Acacieae
47	LN885270	*Acacia fragilis* Maiden & Blakeley	Fabaceae; Caesalpinioideae; mimosoid clade; Acacieae

48	LN885269	*Acacia formidabilis* R.S.Cowan & Maslin	Fabaceae; Caesalpinioideae; mimosoid clade; Acacieae
49	LN885268	*Acacia exocarpoides* W.Fitzg.	Fabaceae; Caesalpinioideae; mimosoid clade; Acacieae
50	LN885266	*Acacia erinacea* Benth.	Fabaceae; Caesalpinioideae; mimosoid clade; Acacieae
51	LN885264	*Acacia eremaea* C.R.P.Andrews	Fabaceae; Caesalpinioideae; mimosoid clade; Acacieae
52	LN885263	*Acacia effusifolia* (R.S. Cowan & Maslin) Maslin & Buscumb	Fabaceae; Caesalpinioideae; mimosoid clade; Acacieae
53	LN885261	*Acacia duriuscula* W.Fitzg.	Fabaceae; Caesalpinioideae; mimosoid clade; Acacieae
54	LN885260	*Acacia diallaga* Maslin & Buscumb	Fabaceae; Caesalpinioideae; mimosoid clade; Acacieae
55	LN885259	*Acacia daphnifolia* Meissner	Fabaceae; Caesalpinioideae; mimosoid clade; Acacieae
56	LN885258	*Acacia cyclops* G.Don	Fabaceae; Caesalpinioideae; mimosoid clade; Acacieae
57	LN885257	*Acacia coolgardiensis* Maiden	Fabaceae; Caesalpinioideae; mimosoid clade; Acacieae
58	LN885256	*Acacia colletioides* Benth.	Fabaceae; Caesalpinioideae; mimosoid clade; Acacieae
59	LN885255	*Acacia cerastes* Maslin	Fabaceae; Caesalpinioideae; mimosoid clade; Acacieae
60	LN885254	*Acacia burkittii* Benth.	Fabaceae; Caesalpinioideae; mimosoid clade; Acacieae
61	LN885252	*Acacia blakelyi* Maiden	Fabaceae; Caesalpinioideae; mimosoid clade; Acacieae
62	LN885251	*Acacia aulacophylla* R.S.Cowan & Maslin	Fabaceae; Caesalpinioideae; mimosoid clade; Acacieae
63	LN885250	*Acacia assimili*s S.Moore subsp. *assimilis*	Fabaceae; Caesalpinioideae; mimosoid clade; Acacieae
64	LN885248	*Acacia ashbyae* Maslin	Fabaceae; Caesalpinioideae; mimosoid clade; Acacieae
65	LN885247	*Acacia anthochaera* Maslin	Fabaceae; Caesalpinioideae; mimosoid clade; Acacieae
66	LN885245	*Acacia andrewsii* W.Fitzg.	Fabaceae; Caesalpinioideae; mimosoid clade; Acacieae
67	LN885244	*Acacia ampliata* R.S.Cowan & Maslin	Fabaceae; Caesalpinioideae; mimosoid clade; Acacieae
68	LN885243	*Acacia acuminata* Benth.	Fabaceae; Caesalpinioideae; mimosoid clade; Acacieae
69	LN885241	*Acacia acuaria* W.Fitzg.	Fabaceae; Caesalpinioideae; mimosoid clade; Acacieae
70	LN885240	*Acacia acanthoclada* F.Muell. subsp. *glaucescens* Maslin	Fabaceae; Caesalpinioideae; mimosoid clade; Acacieae
71	NC_036736	*Senegalia laeta* (R.Br. ex Benth.) Seigler & Ebinger	Fabaceae; Caesalpinioideae; mimosoid clade; Acacieae
72	NC_036735	*Vachellia seyal* (Delile) P.J.H.Hurter	Fabaceae; Caesalpinioideae; mimosoid clade; Acacieae
73	NC_036734	*Vachellia flava* (Forssk.) Kyal. & Boatwr.	Fabaceae; Caesalpinioideae; mimosoid clade; Acacieae
74	KY100266	*Vachellia tortili*s (Forssk.) Galasso & Banfi subsp. *raddiana* (Savi) Kyal. & Boatwr.	Fabaceae; Caesalpinioideae; mimosoid clade; Acacieae
75	KY100264	*Vachellia nilotica* (L.) P.J.H.Hurter & Mabb. subsp. *tomentosa* (Benth.) Kyal. & Boatwr.)	Fabaceae; Caesalpinioideae; mimosoid clade; Acacieae
76	NC_034987	*Albizia odoratissima* (L.f.) Benth.	Fabaceae; Caesalpinioideae; mimosoid clade; Ingeae

77	NC_034988	*Archidendron lucyi* F.Muell.	Fabaceae; Caesalpinioideae; mimosoid clade; Ingeae
78	NC_035347	*Faidherbia albida* (Delile) A.Chev.	Fabaceae; Caesalpinioideae; mimosoid clade; Ingeae
79	NC_028732	*Inga leiocalycina* Benth.	Fabaceae; Caesalpinioideae; mimosoid clade; Ingeae
80	NC_035348	*Pararchidendron pruinosum* (Benth.) I.C.Nielsen	Fabaceae; Caesalpinioideae; mimosoid clade; Ingeae
81	LN885334	*Paraserianthes lophantha* (Willd.) I.C.Nielsen subsp. *lophantha*	Fabaceae; Caesalpinioideae; mimosoid clade; Ingeae
82	NC_034991	*Pithecellobium flexicaule* (Benth.) J.M.Coult.	Fabaceae; Caesalpinioideae; mimosoid clade; Ingeae
83	NC_034992	*Samanea saman* (Jacq.) Merr.	Fabaceae; Caesalpinioideae; mimosoid clade; Ingeae
84	NC_034986	*Adenanthera microsperma* Teijsm. & Binn.	Fabaceae; Caesalpinioideae; mimosoid clade; Mimoseae
85	NC_035346	*Dichrostachys cinerea* (L.) Wight & Arn.	Fabaceae; Caesalpinioideae; mimosoid clade; Mimoseae
86	NC_028733	*Leucaena trichandra* (Zucc.) Urb.	Fabaceae; Caesalpinioideae; mimosoid clade; Mimoseae
87	NC_034989	*Parkia javanica* (Lam.) Merr.	Fabaceae; Caesalpinioideae; mimosoid clade; Mimoseae
88	NC_034990	*Piptadenia communis* Benth.	Fabaceae; Caesalpinioideae; mimosoid clade; Mimoseae
89	KJ468101	*Prosopis glandulosa* Torr.	Fabaceae; Caesalpinioideae; mimosoid clade; Mimoseae
90 #	KJ468096	*Ceratonia siliqua* L.	Fabaceae; Caesalpinioideae; Umtiza clade;
91	NC_036761	*Adenolobus garipensis* (E.Mey.) Torre & Hillc.	Fabaceae; Cercidoideae; Cercideae
92 #	MF741770	*Cercis chuniana* F.P.Metcalf	Fabaceae; Cercidoideae; Cercideae
93 #	KF856619	*Cercis canadensis* L.	Fabaceae; Cercidoideae; Cercideae
94	NC_036744	*Daniellia pilosa* (J.Léonard) Estrella	Fabaceae; Detarioideae
95	NC_036743	*Crudia harmsiana* De Wild.	Fabaceae; Detarioideae; Detarieae
96	NC_036742	*Guibourtia leonensis* J.Léonard	Fabaceae; Detarioideae; Detarieae
97 #	KJ468103	*Tamarindus indica* L.	Fabaceae; Detarioideae; Detarieae
98* #	DQ119058	*Cucumis sativus* L.	Cucurbitaceae; Benincaseae
